# DDX6 interacts with DDX3X to repress translation in microRNA-mediated silencing

**DOI:** 10.1093/nar/gkaf868

**Published:** 2025-09-09

**Authors:** Yanyan Lu, Meng Tao, Hong Su, Yiren Tu, Ji-Ping Wang, Masahiko Kuroda, Xiaozhong Wang

**Affiliations:** Department of Molecular Biosciences, Northwestern University, Evanston, IL 60208, United States; Department of Molecular Biosciences, Northwestern University, Evanston, IL 60208, United States; Department of Molecular Biosciences, Northwestern University, Evanston, IL 60208, United States; Department of Molecular Biosciences, Northwestern University, Evanston, IL 60208, United States; Department of Statistics and Data Science, Northwestern University, Evanston, IL 60208, United States; Department of Molecular Pathology, Tokyo Medical University, 6-1-1, Shinjuku, Shinjuku-ku 160-8402, Tokyo, Japan; Department of Molecular Biosciences, Northwestern University, Evanston, IL 60208, United States

## Abstract

DDX6 is known to repress messenger RNA (mRNA) translation and promote mRNA decay in microRNA-mediated silencing. In embryonic stem cells (ESCs), DDX6 primarily functions at the translation level, independent of mRNA destabilization; however, the precise molecular mechanism of how DDX6 represses translation remains unclear. Here, we identify DDX3X as a key downstream target of DDX6-mediated translational repression in ESCs. Conditional knockout of DDX3X demonstrates its essential role in microRNA (miRNA) silencing. Biochemical analyses reveal that DDX6 directly binds to DDX3X, with the C-terminal region of DDX6 being necessary for this interaction. ESCs lacking DDX6 and rescued with a DDX6 mutant that is defective in DDX3X interaction continue to exhibit miRNA silencing defects. Furthermore, the mutant DDX6 is unable to inhibit 48S preinitiation complex formation *in vitro*. These findings uncover a novel mechanism in which DDX6 represses target mRNA translation via its interaction with DDX3X.

## Graphical Abstract

**Graphical Abstract:**
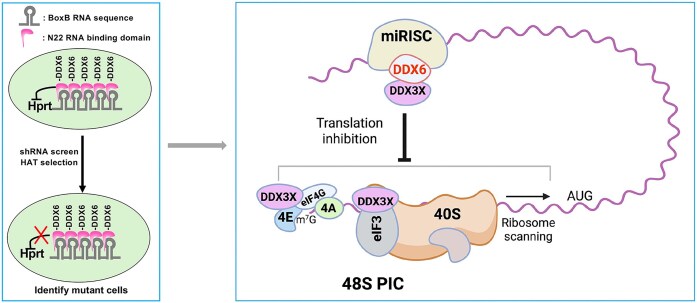

## Introduction

MicroRNAs (miRNAs) are a class of small, noncoding RNAs that act as molecular rheostats, fine-tuning the protein output of individual transcripts [1]. With 1917 annotated human miRNAs [2], it is estimated that they collectively regulate over 60% of all protein-coding genes in the genome [3, 4]. As a result, miRNAs are implicated in nearly all developmental and pathophysiological processes [5–8]. To carry out their regulatory functions, miRNAs bind to Argonaute (Ago) proteins, forming the core of the miRNA-induced silencing complex (miRISC) [9–11]. Through conserved scaffold proteins TNRC6/GW182, Ago recruits additional protein effectors to assemble fully functional miRISCs at their messenger RNA (mRNA) targets [12, 13]. In animals, miRISCs regulate gene expression through two main pathways: translational repression and mRNA degradation [11]. The mechanism by which miRNAs promote mRNA degradation is well understood. In this pathway, TNRC6/GW182 proteins play a pivotal in recruiting degradation machinery to miRISCs [14]. The degradation process begins with deadenylation by the PAN2-PAN3 and CCR4-NOT complexes, which shortens the poly(A) tail. Next, the 5′ cap is removed by the DCP1–DCP2 decapping complex. Finally, the decapped mRNA is degraded by the XRN1 5′–3′ exonuclease.

In contrast, the molecular details of miRNA-mediated translational repression remain less defined despite extensive research [11, 14–16]. Current evidence suggests that miRNAs inhibit cap-dependent mRNA translation during the initiation stage [17–22]. Proposed models implicate the CCR4-NOT complex and its associated proteins in displacing key translation initiation factors—such as eIF4E, eIF4G, and eIF4A—thereby preventing the formation of the 48S pre-initiation complex (PIC) [11]. However, the precise molecular mechanisms remain an area of active investigation due to several challenges. First, while global studies suggest that mRNA decay is the primary mode of miRNA silencing, translational repression often precedes mRNA deadenylation, with mRNA decay becoming more prominent over time [23–26]. This dynamic interplay between the two processes makes it difficult to isolate the contribution of a single mechanism. Second, miRISCs are composed of various protein complexes with overlapping or redundant functions, complicating the assessment of their individual roles within a single pathway. For example, the CCR4-NOT complex serves both as an essential deadenylase and a hub for recruiting translational repressors [27–32]. Third, the mode of miRNA silencing varies across developmental stages. For example, in early embryos, miRNAs primarily induce translational repression with minimal effects on mRNA stability [33, 34], whereas mRNA destabilization becomes more widespread in post-embryonic cells [23, 24, 35, 36]. These context-dependent variations present additional challenges in defining a universal mechanism.

Central to miRNA-mediated silencing is the downstream effector protein DEAD-box helicase 6 (DDX6) [37]. DDX6 interacts with multiple components within miRISCs, with CNOT1, the large scaffold of the CCR4-NOT complex, playing a key role in recruiting DDX6 to miRISCs [30, 38, 39]. Mechanistically, DDX6 can repress translation independently of mRNA degradation, as well as activate decapping to promote mRNA decay [40, 41]. These conserved functions of DDX6 are thought to converge to ensure efficient miRISC-dependent gene silencing [11, 14]. For mRNA decapping, DDX6 directly interacts with the decapping activators PATL1, EDC3, and LSM14A to assemble the decapping complex, leading to DCP2 activation [38, 39, 42]. For translational repression, the yeast ortholog of DDX6, Dhh1p, has been shown to repress global translation at the initiation stage in response to nutrient deprivation [40]. In animal cells, DDX6 depletion suppresses miRNA-mediated translation inhibition, as well as the silencing caused by tethering miRISC components such as Ago, TNRC6/GW182, and CNOT1 [30, 37–39, 43]. Recent studies have found that DDX6 interacts with the eIF4E transporter (4E-T), an eIF4E-binding protein [28, 29, 44]. Experimental evidence suggests that 4E-T can act downstream of DDX6 to repress translation initiation, either through direct competition for eIF4G or indirect competition for the 5′ cap via 4EHP [11]. However, 4E-T depletion only partially alleviates miRNA silencing [44], indicating that DDX6 may utilize additional, as-yet-unknown binding partners in translational regulation [11, 15].

Our study aims to explore how DDX6 represses translation. Recent global analyses of mRNA stability and translation efficiency in embryonic stem cells (ESCs) reveal that miRNAs and DDX6 predominantly regulate their targets through translational repression, independent of mRNA destabilization [45]. These findings in ESCs parallel observations from early embryonic development [33, 34], making ESCs an ideal system to investigate the molecular mechanisms of DDX6-mediated translational repression without the confounding effects of mRNA degradation. To identify new downstream factors of DDX6, we designed a drug-selection-based RNA interference (RNAi) screen in ESCs, leveraging previous findings that tethering DDX6 to a reporter mRNA inhibits its translation [15, 37, 43, 46]. From this screen, we identified DDX3X as a critical downstream target of DDX6 in ESCs. Like DDX6, DDX3X is essential for translational silencing. Moreover, mutant DDX6 unable to bind DDX3X fails to repress translation both *in vivo* and *in vitro*. These results demonstrate that the interaction between DDX6 and DDX3X is a crucial step for miRISC to inhibit translation initiation.

## Materials and methods

### Cell culture

COS-7 (CRL-1651) and 293T (CRL-3216) cells were originally purchased from the American Type Culture Collection (ATCC) and cultured at 37°C with 5% CO_2_ in Dulbecco’s modified Eagle’s medium (DMEM; Gibco) supplemented with 10% fetal bovine serum (FBS), penicillin–streptomycin–glutamine (Thermo Fisher Scientific, 10378016, 100 units/ml). The mouse ESCs (AB2.2) and its derivative cell lines were originally obtained from Allan Bradley’s lab [47] and cultured in KnockOut™ DMEM (Thermo Fisher Scientific, 10829018) with 15% FBS and mLIF. All cell lines were confirmed to be mycoplasma-free using the LookOut^®^ Mycoplasma polymerase chain reaction (PCR) Detection Kit (Sigma, MP0035).

### Construction of mESC reporter lines

The 1A2 mESCs that expressed both the control firefly luciferase (Ff-Luc) and the reporter renilla luciferase (Rn-Luc), with five copies of Box B in the 3′UTR, were generated through hypoxanthine–aminopterin–thymidine (HAT) selection following the electroporation of linearized *CMV-Ff-Luc-bPA* and *CMV-Rn-Luc-5BoxB-bPA::PGK-Hprt* plasmids, as previously described [43]. To investigate DDX6-mediated translational repression, *PB-CAG-λN22HA-DDX6::PGK-Puro* was integrated into 1A2 cells via PiggyBac (PB)-mediated transposition [48]. Stable cell lines, including 1D1, were established through Puro selection. To establish a drug-selectable reporter ES cell line, a linearized *PGK-Hprt-5BoxB-bPA::PGK-Neo-bPA* vector was electroporated into an AB2.2-derived mESC line [43], resulting in a stable line named “7–22”, which was obtained through G418 and HAT selection. Subsequently, λN22-DDX6 was overexpressed using *PB-CAG-λN22HA-DDX6::PGK-Hyg*. Following hygromycin B (Hyg) and 6-thioguanine (6-TG) selection, the 1G1 reporter cell line was chosen for subsequent RNAi screens.

### shRNA (short hairpin RNA) screen

A custom-made pooled retroviral pSM2-shRNA-mir30 library was purchased from Open Biosystems [49]. Using Lipofectamine 2000, we transiently transfected an individual shRNA plasmid pool into retroviral packaging Phoenix cells to produce recombinant retroviruses [43]. Briefly, Phoenix cells were plated on a 10-cm cell culture dish at a confluency of 60%. For each pool, 5 μg of library DNA was transfected into 8 ml of Opti-MEM media. The supernatant containing recombinant retroviruses was harvested 48 h post-transfection by filtering through a 0.45-μm filter. The 1G1 reporter ES cells were infected with the retroviral library and sequentially selected with puromycin (2 μg/ml), followed by HAT selection to isolate single HAT+ colonies. Individual clones were expanded to confirm drug resistance and to isolate genomic DNA. Candidate shRNAs were identified through sequence analysis of PCR-amplified proviral fragments encoding gene-specific shRNAs. After screening over 40 000 independently lentivirus-transduced mESC colonies, we obtained and analyzed 43 different candidate colonies. The shRNA targets are listed in Supplementary Table S1.

### Gene targeting in mESCs

To generate DDX6 or DDX3X conditional knockout (cKO) mESCs, we obtained the gene-targeting vectors *PG00193_Z_8_C01* for DDX6 and *PG00217_Z_5_A01* for DDX3X from the European Conditional Mouse Mutagenesis Program (EUCOMM) [50]. Male mouse AB2.2 ES cells were electroporated with linearized DNA, and drug-resistant single clones were selected as previously reported [51]. Long-range PCR was used to identify 5′- and 3′-homologous recombination events. For DDX6, following successful targeting of the first allele, the drug selection cassette was excised via transient transfection of FLP recombinase, leaving only the LoxP-flanked exon. Targeting of the second allele and removal of its drug selection cassette were performed similarly. For DDX3X, only one round of targeting was required due to its X-linked nature. To generate 4-hydroxytamoxifen (4-OHT)-inducible knockout mESCs, we introduced either the *PB-CAG-ERT2-Cre-ERT2::PGK-Hygro* or *PB-CAG-ERT2-Cre-ERT2::PGK-Puro* vector. Primer sequences used for PCR screening are listed in Supplementary Table S2.

### Complementary DNA expression vectors

All complementary DNA (cDNA) expression vectors for stable transfection were constructed as PB vectors with the CAG promoter to express various genes of interest and the mouse PGK promoter for a drug resistance gene [51]. Various point mutation or truncated expression vectors for DDX6 or DDX3X were constructed using overlapping PCR. All related primers are listed in Supplementary Table S2. All expression vectors were verified through restriction enzyme digestion and sequencing analysis.

### Reverse transcription and quantitative PCR analysis

Total RNA from cells was purified using TRIzol (Thermo Fisher Scientific, 15596018) followed by DNase I (Thermo Fisher Scientific, AM2222) digestion. Reverse transcription was performed on 2 μg of total RNA using Superscript™ III Reverse Transcriptase (Thermo Fisher Scientific, 18080085) with oligo-dT, random primers, or gene-specific primers. Quantitative PCR was conducted with Power SYBR Green PCR Master Mix (Thermo Fisher, 4368702) using an ABI PRISM 7700 Sequence Detection System or QuantStudio™ 3 Real-Time PCR System. GAPDH or Ff-Luc mRNA was used as an internal control. Primers used in the experiments are listed in Supplementary Table S2. Relative expression levels of the target gene were calculated using the ΔΔCT method. Data are presented as fold changes in mRNA levels relative to the control cells, with values defined as 1.

### Measurement of the rate of global protein synthesis

The rate of protein synthesis was measured using a nonradioactive SUNSET method [52]. Cells were transiently labeled with puromycin at a final concentration of 2 μg/ml for 5 min. An anti-puromycin antibody (KeraFAST, 3RH11) was used to detect labeled proteins via western blot.

### Subcellular localization of DDX6 and DDX3

For immunofluorescence and confocal imaging analysis, mESCs or COS-7 cells were cultured on gelatin-coated four-well chambered coverglass. After washing with phosphate-buffered saline (PBS), the cells were fixed with 2% paraformaldehyde for 10 min. The fixed cells were rinsed with PBS three times and then incubated in blocking buffer (10% heat inactivated goat serum, 10% heat inactivated donkey serum, 0.1% Triton X-100 in PBS) for 30 min. After blocking, the slides were incubated with a primary antibody diluted in blocking buffer overnight at 4°C. The slides were then washed five times with PBST (PBS containing 0.2% Tween 20), followed by incubation with a fluorescently conjugated secondary antibody diluted in blocking buffer for 1 h. After five washes with PBST, the slides were incubated with 4′,6-diamidino-2-phenylindole (DAPI) at 0.5 μg/ml for 5 min, then mounted with Vectashield antifade mounting medium (Vector Laboratories, H-1000). Images were captured using a Zeiss LSM-710 laser scanning confocal microscope.

### miRNA reporter and dual luciferase assays

The mir-CXCR reporter and dual luciferase assays were performed as previously described [43, 53]. Briefly, mESCs were seeded into 24-well plates and transfected using Lipofectamine 2000. For the mir-CXCR assay, each well received 0.1-μg *CMV-Ff-Luc*, 0.02-μg *CMV-Rn-Luc-CXCR*, and 7.5-nM double-stranded RNA (dsRNA). mir-30 dsRNA was used as a negative control. Dual luciferase activity was measured using the Dual-Luciferase Reporter Assay System (Promega, E1980). For ease of comparison, the dual luciferase ratio from control groups was normalized to 1. Fold repression in this miRNA reporter assay was defined as the ratio of the dual-luciferase signal from the control group (mir-30) to that of the experimental group. For the Bim 3′UTR reporter assay [53, 54], feeder-free DDX6 cKO mESCs were cultured with or without 4-OHT for 2 days prior to transfection. On the day of transfection, 2 × 10^5^ suspended cells per well were seeded in 24-well plates and transfected using FuGene HD Transfection Reagent (Promega, E2311). Each well received 0.2 μg of CMV-Ff-Luc and 0.025 μg of either CMV-Rn-Luc-Bim-3′UTR-WT or -Mut plasmids. Transfected cells were maintained in the presence or absence of 4-OHT and harvested for dual-luciferase assays 2 days post-transfection. Fold repression was calculated as the ratio of normalized Renilla luciferase activity between mutant and wild-type Bim-3′UTR reporters.

### Recombinant protein expression and purification

DDX6 and DDX3X cDNA were cloned into *pGEX-KG* for GST-fusion protein expression, *pET28* for 6xHis protein expression, and *pMCSG9* for 6xHis-MBP-fusion protein expression. Due to the poor solubility of full-length DDX3X in bacteria, a truncated construct encoding 6xHis-MBP-DDX3X^166-590^, encompassing the full helicase domain, was used for expression and purification in *Escherichia coli*.

BL21(DE3) transformants were cultured in 100-ml Luria Broth (LB) medium supplemented with the appropriate antibiotic (ampicillin 100 μg/ml or kanamycin 50 μg/ml) at 37°C until OD_600_ reached 0.8. Protein expression was induced by the addition of isopropyl-β-D-thiogalactopyranoside to a final concentration of 0.25 mM. Cultures were shifted to 18°C and incubated for an additional 12 h before harvesting by centrifugation. Cell pellets were resuspended in 1/10 of the culture volume in TNE buffer [20-mM Tris–HCl, pH 8.0, 100-mM NaCl, 1-mM Phenylmethylsulfonyl fluoride (PMSF)], then frozen at −80°C overnight. The next day, pellets were thawed on ice, and lysozyme was added to a final concentration of 100 μg/ml. After shaking for 20 min in the cold room, Triton X-100 was added to a final concentration of 1%. The lysate was incubated on ice for 10 min until the solution clarified. The preparation was then diluted with 30-ml PBS-450T without ethylenediaminetetraacetic acid (EDTA) [450-mM NaCl, 20-mM Na_2_HPO_4_, pH 7.3, 1-mM PMSF, 1-mM Dithiothreitol (DTT), 1% Triton X-100], rotated in the cold room for 30 min, and briefly sonicated on ice. Lysates were clarified by centrifugation at 25 000 rpm for 30 min at 4°C, and the supernatant was filtered through a 0.45-μm syringe filter, if necessary. For affinity purification, 0.5 ml of Glutathione Sepharose 4B resin (GE Healthcare, 17075601) or HisPur™ Ni-NTA resin (Thermo Scientific, 88221) was equilibrated in 5-ml PBS-450T. The cleared lysate was incubated with the resin by rotation in the cold room for 1 h. After binding, the mixture was centrifuged at 2000 rpm for 5 min at 4°C, and the supernatant was discarded. Beads were washed three times with 35-ml PBS-450T, followed by two washes in PBST (150-mM NaCl, 20-mM Na_2_HPO_4_, pH 7.4, 0.5-mM EDTA, 1-mM PMSF, 0.01% Triton X-100). Washed beads were stored on ice for use in pull-down assays. For protein elution, GST-fusion proteins were eluted using buffer containing 75-mM reduced glutathione, 50-mM NaCl, 1-mM DTT, 1-mM PMSF, and 0.01% Triton X-100, with rotation for 15 min at 4°C. 6 × His-tagged proteins were eluted with buffer containing 500-mM imidazole. Elution was repeated twice, and the combined eluates were dialyzed overnight in PBS. Protein concentration was measured, and purified proteins were stored at −80°C for later use.

### Western blot, immunoprecipitation and GST pull-down

For western blot analyses, cell pellets were washed with PBS and lysed directly in Laemmli buffer [62.5 mM Tris–HCl, pH 6.8, 2.0% sodium dodecyl sulphate (SDS), 10% glycerol, 5% 2-mercaptoethanol, and 0.002% bromophenol blue]. For input lysates in protein–protein interaction assays, cells were first lysed in radio-immunoprecipitation assay (RIPA) buffer (50-mM Tris, pH 7.5, 150-mM NaCl, 1.0% NP-40, 10% glycerol, 1-mM EDTA) supplemented with cOmplete™ protease inhibitor (Sigma, 4693124001). Portions of the lysates were mixed with 2× Laemmli buffer for western blot analysis. Sonicated and boiled protein samples were resolved on 10% sodium dodecyl sulfate–polyacrylamide gels and transferred to Polyvinylidene difluoride (PVDF) membranes. The membranes were blocked with 5% nonfat milk in PBS buffer containing 0.1% Tween 20, followed by incubation with specific primary antibodies and horseradish peroxidase-conjugated secondary antibodies. Signals were visualized using Pierce™ ECL Western Blotting Substrate (Thermo Fisher Scientific, 32106). For quantitative western blotting, membranes were stained with the Revert 700 Total Protein Stain Kit (LI-COR, 926-11010) and imaged on the ODYSSEY CLx system (700-nm channel) for normalization. Following primary antibody incubation, membranes were probed with Goat anti-Rabbit IgG DyLight™ 800 (Invitrogen, SA5-35571) and imaged on the 800-nm channel. A complete list of antibodies used is provided in Supplementary Table S3.

To detect protein–protein interactions, co-immunoprecipitation (co-IP) of protein complexes from mESCs or 293T cells was carried out as previously described [43]. Briefly, mESCs or 293T cells expressing Flag-tagged proteins and their interacting partners were lysed in lysis buffer (50-mM Tris, pH 7.5, 150-mM NaCl, 1.0% NP-40, 10% glycerol, 1-mM EDTA, 1-mM PMSF, and cOmplete™ Mini Protease Inhibitor Cocktail). After clearing the lysates by centrifugation at 14 000 rpm for 10 min, the lysates were incubated with anti-Flag M2 affinity gel (Sigma, A2220) to capture protein complexes. After washing, the affinity beads were eluted in Laemmli buffer for western blot analysis.

For GST pull-down experiments, recombinant GST fusion proteins were purified and immobilized on Glutathione Sepharose 4B beads (GE Healthcare, 17 075 601). Aliquots of immobilized GST fusion proteins (10 μg) were incubated with 293T cell lysates expressing various tagged interactor proteins or purified MBP-fusion proteins in RIPA buffer. After binding and washing, the pulled-down proteins were detected by western blot using tag- or protein-specific antibodies.

For the competitive binding assay of DDX3X between DDX6 and eIF3 experiment, we established two HEK293T cell lines stably expressing Flag-DDX3X and HA-eIF3F, respectively. To affinity-purify Flag-DDX3X, cell pellets were initially extracted with Buffer I (20-mM Tris–HCl, pH 7.4, 0.5% Triton X-100, 5-mM MgCl_2_, 100-mM KCl, 1-mM PMSF, and 1 × EDTA-free protease inhibitor tablet). After removing nuclear debris by low-speed centrifugation, the supernatant was adjusted to Buffer II conditions by adding KCl and Triton X-100 to final concentrations of 450 mM and 1%, respectively. The lysates were then clarified by centrifugation at 14 000 rpm for 20 min at 4°C. The resulting supernatant was diluted to reduce the KCl concentration to 150 mM before performing anti-Flag affinity purification. Following incubation and washes, Flag-DDX3X was immobilized on M2 agarose beads for use in the competitive binding assay. Flag-DDX3X-bound beads were pre-incubated with 10 μg/ml of purified recombinant His-MBP or His-MBP-DDX6 for 30 min. In parallel, lysates from HA-eIF3F-expressing HEK293T cells were prepared using the same protocol as for Flag-DDX3X. These lysates were then added to the pre-incubated Flag-DDX3X beads to assess eIF3 complex binding. Interacting components of the eIF3 complex were detected by western blotting.

### Sucrose gradient ultracentrifugation analysis

For polysome fractionation analysis, cells were cultured in 15-cm culture dishes, treated with 100 μg/ml cycloheximide for 5 min, and washed with PBS containing 100 μg/ml cycloheximide before harvesting. A total of 2 × 10^7^ cells were scraped into 1 ml of polysome extraction buffer (10-mM Tris–HCl, pH 7.4, 0.5% Triton X-100, 15-mM MgCl_2_, 100 μg/ml cycloheximide, 0.2-M NaCl, 1-mM PMSF, 20 U/ml RNasin, 1 mg/ml heparin, 1× EDTA-free protease inhibitor tablet). Cell lysates were incubated on ice for 10 min and passed through a 21-gauge syringe 10 times. Nuclei and debris were removed by centrifugation at 12 000 × *g* for 10 min at 4°C. A total volume of 400 μl of supernatant, equivalent to 20 OD_260_, was subjected to 11-ml sucrose gradients (10%–35%) with 500 μl of 70% sucrose cushion at the bottom. Ultracentrifugation was carried out using a SW41 rotor (Beckman Coulter) at 36 000 rpm for 2 h at 4°C. After centrifugation, each sample was separated into 12 equal fractions while absorbance at 254 nm was continuously monitored using a Biologic LP chromatography system (Bio-Rad, 731-8300). To recover RNA, each fraction or pooled fractions were incubated with SDS (final concentration 1%) and proteinase K (10 μg/ml) for 30 min at 42°C, extracted with an equal volume of phenol/chloroform/isoamyl alcohol, and precipitated with ethanol. RNA pellets from each fraction were resuspended in RNase-free ddH_2_O, treated with DNase I, and purified for Reverse Transcription quantitative Polymerase Chain Reaction (RT-qPCR)analysis. To collect total proteins, fractions were precipitated with trichloroacetic acid at a final concentration of 10% (w/v) and incubated at 4°C for 30 min. After centrifugation at 10 000 × *g* for 10 min at 4°C, protein pellets were washed three times with cold acetone and resuspended in SDS Laemmli buffer for western blot analysis.

### *In vitro* transcription

To prepare capped mRNA for *in vitro* translation, we generate DNA templates that consist of T7 promoter and poly(A) tail by PCR amplification. The Rn-Luc mRNA contains a 235-nt 5′UTR from the mouse Bim gene. The primer sequences are included in Supplementary Table S2. PCR-generated DNA templates were purified by agarose gel electrophoreses. *In vitro* transcription using 1 μg of the DNA template in 20 μl reaction volume was carried out with MAXIscript T7 Transcription Kit (Thermo Fisher Scientific, AM1314M) in the presence of m^7^GpppG cap analog (NEB, S1405S). mRNA products were treated with DNase I to remove DNA templates and purified with RNA Clean & Concentrator^TM^-5 (Zymo Research, R1015).

### *In vitro* protein translation

*In vitro* translation was performed using the Rabbit Reticulocyte Lysate system (Promega, L4960) according to the manufacturer’s protocol. Purified Ff-Luc and Rn-Luc mRNAs were first denatured at 65°C for 3 min before setting up a 50 μl reaction. Reactions were incubated at 30°C for 20, 40, and 80 min. In the presence of purified recombinant MBP, MBP-DDX6, or MBP-DDX6^Mut6^ at a final concentration of 0.1 μM or 0.25 μM, translation reactions were incubated at 30°C for 20, 40, and 80 min. To measure the translation levels of both Ff-Luc and Rn-Luc, 10 μl of reaction was sampled at indicated time points using a luciferase activity assay (Promega, E1980). For analysis of translation PIC, GMP-PNP was added to a final concentration of 2 mM. Purified MBP, MBP-DDX6, and MBP-DDX6^Mut6^ proteins were added to reactions at a final concentration of 0.25 μM, respectively. *In vitro* translation reactions were then analyzed by 5%–20% sucrose gradient ultracentrifugation.

### Kinetic measurement of protein–protein interaction

Purified recombinant 6xHis-MBP-DDX3X^166-590^ and GST-DDX6 fusion proteins were used to measure the binding affinity between DDX6 and DDX3X using a BLItz Bio-layer Interferometer (ForteBio) at the Keck Biophysics Facility of Northwestern University. The Ni-NTA biosensors were first soaked in BLItz assay buffer (20-mM Tris–HCl, pH 8.0, 150-mM KCl, 0.02% Tween 20, 2-fmM DTT, 1 mg/ml bovine serum albumin) and then loaded with 6xHis-DDX3X proteins. After washing with BLItz assay buffer, the loaded Ni-NTA sensor tips were incubated in solutions containing GST, GST-DDX6, or GST-DDX6^Mut6^ at concentrations of 125, 250, and 500 nM to measure complex formation. Sensorgrams were globally fit to a 1:1 binding model, and the equilibrium dissociation constant was calculated.

### Quantification and statistical analysis

All data are presented as means with standard error of the means. Comparisons between two groups are analyzed using two-tailed unpaired Student’s *t-*test. *P <.05, **P <.01, ***P <.001, compared to the control groups. All measurements were performed in triplicate.

## Results

### miRNA silencing defects in DDX6 cKO ESCs

To investigate the function of DDX6, we generated a conditional DDX6 knockout mESC line. Using a targeting vector designed by the European Conditional Mouse Mutagenesis Program (EUCOMM) [50] (Supplementary Fig. S1A), we obtained DDX6^flox/flox^ homozygous mESCs. In these DDX6 cKO ESCs (*DDX6^flox/flox^; CAG-ER^T2^-Cre-ER^T2^*), DDX6 protein was depleted following 4-OHT treatment, as confirmed by western blot analysis (Supplementary Fig. S1B). Despite the depletion of DDX6, global protein synthesis rates, assessed via transient puromycin incorporation into nascent polypeptides, remained largely unchanged (Supplementary Fig. S1C). Consistent with previously reported growth defects in DDX6 knockout mESCs [45], DDX6 cKO ES cells exhibited significantly slower growth following DDX6 deletion, compared to 4-OHT-untreated controls or DDX6^flox/flox^ cells lacking Cre expression (Supplementary Fig. S1D). This growth impairment is in line with prior observations in ES cells with disrupted miRNA silencing pathways [53, 55–57].

**Figure 1.**
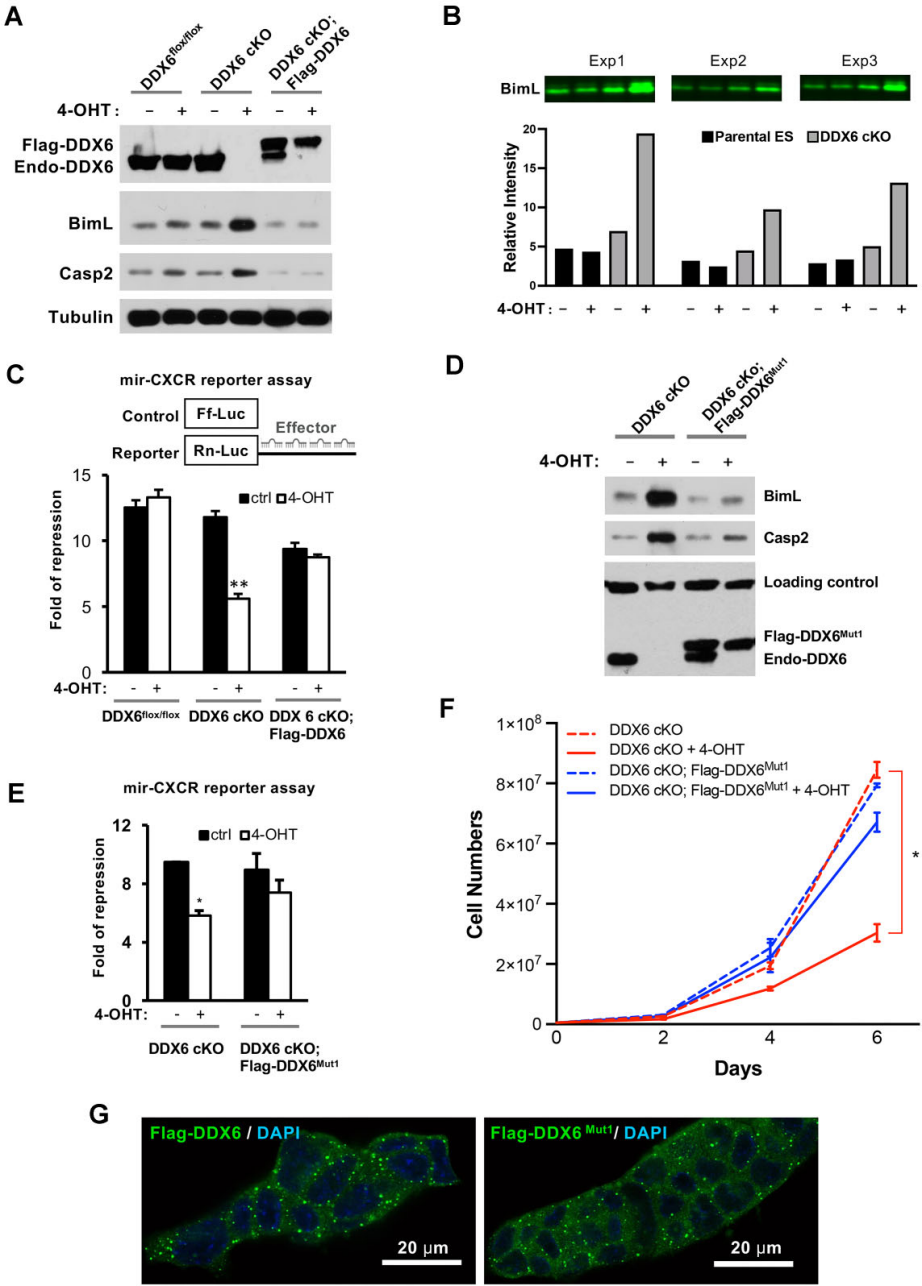
Genetic rescues of miRNA silencing defects in DDX6 cKO mESCs. (**A**) W**e**stern blot analysis showing that Bim and Casp2, two known endogenous miRNA targets in mESCs, were significantly upregulated 4 days after 4-OHT-induced deletion of DDX6. This upregulation was rescued by expression of Flag-tagged DDX6. (**B**) Quantitative western blot analysis confirmed a 1- to 3-fold increase in Bim protein levels in DDX6 cKO mESCs across three independent experiments. Relative Bim intensities were normalized to total protein using Revert 700 total protein stain. (**C**) The mir-CXCR reporter assay revealed a defect in exogenous miRNA silencing in DDX6 cKO mESCs, which was rescued by wild-type Flag-DDX6. (**D, E**). A previously reported mutant, Flag-DDX6^Mut1^, was able to rescue both endogenous (**D**) and exogenous (**E**) miRNA silencing defects. (**F**) Like wild-type DDX6, Flag-DDX6^Mut1^ also rescued the proliferation defect observed in DDX6 cKO mESCs. (**G**) Confocal imaging of genetically rescued mESCs showed that Flag-DDX6^Mut1^ exhibited normal P-body localization. Results in panels (C), (E), and (F) are presented as mean ± standard error of the mean (SEM) from three independent transfections. *P<.05, **P<.01, ***P <.001.

To validate the role of DDX6 in endogenous miRNA silencing, we examined the protein levels of Bim and Casp2, two previously characterized miRNA targets in ESCs [43, 53, 54, 58, 59]. In the absence of DDX6, both Bim and Casp2 were upregulated at the protein level (Fig. 1A). Bim protein levels increased 1- to 3-fold in DDX6 cKO mESCs, as determined by three independent western blot experiments (Fig. 1B). Notably, no increase in Bim or Casp2 mRNA levels was observed (Supplementary Fig. S2A), indicating that their regulation by miRNA and DDX6 is primarily mediated at the level of translation rather than mRNA abundance. Previous studies have shown that miR-19 and miR-92 bind to the 3′UTR of Bim mRNA and repress its translation [53, 54]. Using a previously described Bim-3′UTR reporter assay [53, 54], we demonstrated that endogenous miRNAs suppress Bim-3′UTR reporter expression in a DDX6-dependent manner (Supplementary Fig. S2B). This finding is consistent with results from a recent global study [45]. Using an exogenous mir-CXCR dual luciferase reporter assay [53, 60], we further confirmed that DDX6 cKO ESCs exhibited a miRNA silencing defect (Fig. 1C). Importantly, in both endogenous and exogenous miRNA silencing assays, reintroducing wild-type Flag-tagged DDX6 completely rescued the observed silencing defects (Fig. 1A and C). Unlike previous studies utilizing RNAi-mediated knockdown of DDX6 [38, 39], the DDX6 cKO ESCs provide a null model, enabling the use of genetic rescue experiments to gain mechanistic insights into DDX6’s function.

DDX6 interacts with multiple effector components of the miRISCs. The structural basis for DDX6’s interaction with some of its partners has been determined. For instance, EDC3, LSM14, PATL1, and 4E-T all bind to a common interface on the DDX6 RecA2-C domain in a mutually exclusive manner [11, 29]. A well-characterized mutant DDX6 protein (DDX6^Mut1^), containing multiple alanine substitutions at this interface, is unable to bind to these partner proteins [15, 29, 42]. To investigate whether the interactions between DDX6 and these effector proteins are critical for miRNA silencing in ESCs, we performed a genetic rescue by expressing DDX6^Mut1^ in DDX6 cKO ESCs. DDX6^Mut1^ proteins fully rescued the defects in translational repression, as assessed by both endogenous and exogenous miRNA targets (Fig. 1D and E). Moreover, DDX6^Mut1^ proteins, which localized to P bodies similarly to wild-type DDX6, rescued the proliferation defect of DDX6-null cells (Fig. 1F and G). Thus, our findings support the previous hypothesis that other currently unknown DDX6 partners contribute to DDX6-mediated translational inhibition in ESCs [15]. This discrepancy between our results on DDX6^Mut1^ and some previous findings may be due to differences in cell types or experimental assays. Nonetheless, this observation prompted us to identify new downstream targets of DDX6 involved in translational silencing using ESCs.

### DDX3X as a downstream target for DDX6 mediated translation repression

To explore unknown DDX6 downstream partners, we first established an ESC line stably expressing a Rn-Lucreporter containing five BoxB sites in its 3′UTR. Consistent with previous reports [15, 37, 43, 46], tethering of λN22-DDX6 was sufficient to inhibit Rn-Lucprotein synthesis in stably transfected mESCs without affecting mRNA stability (Supplementary Fig. S3).

**Figure 2.**
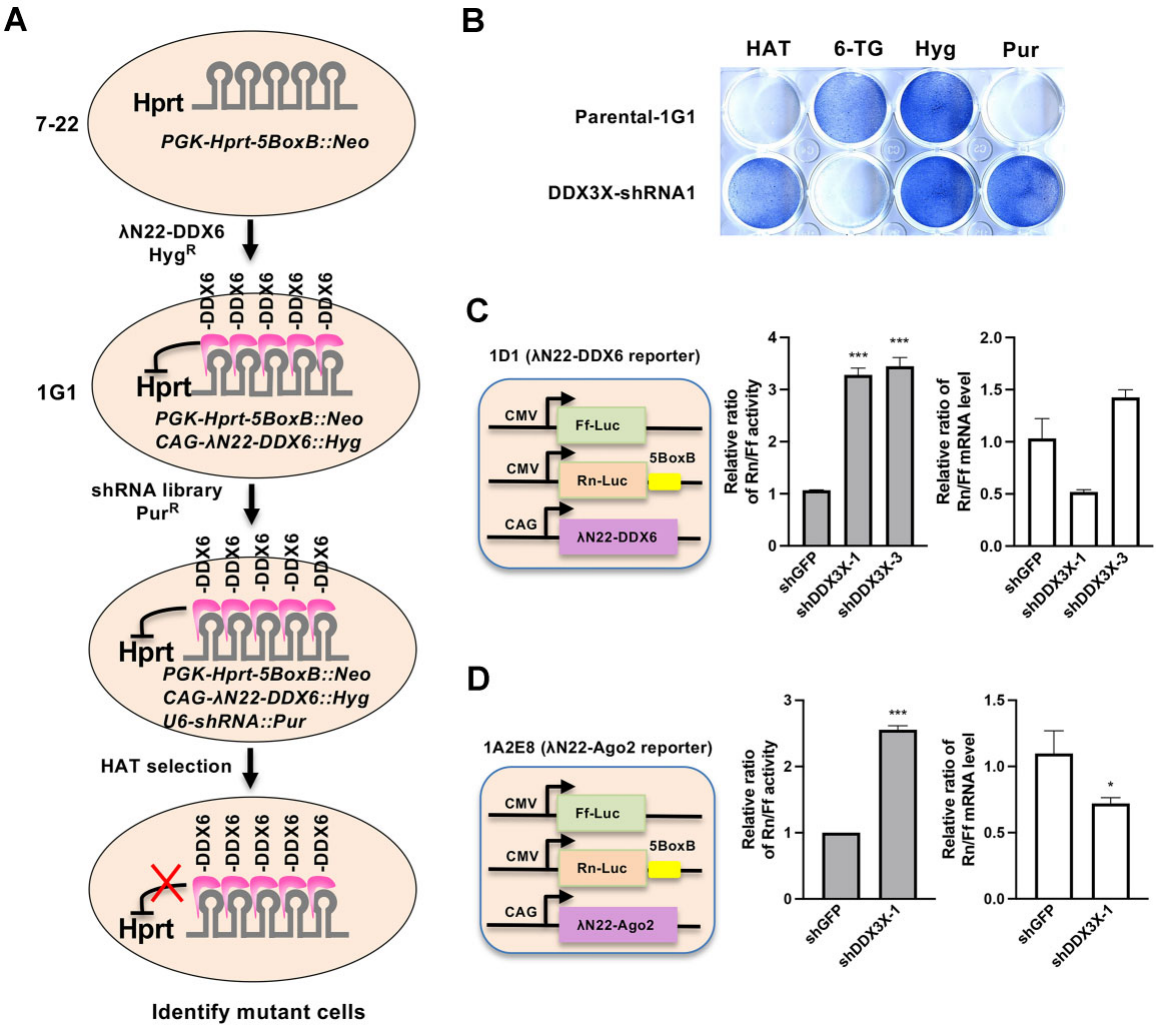
An RNAi screen identified DDX3X as acting downstream of DDX6. (**A**) Experimental design of a drug selection-based shRNA screen to identify genes required for λN22HA-DDX6-mediated silencing of the Hprt-5BoxB reporter gene. The reporter gene and effector PB vectors were sequentially introduced into mESCs, with selection mediated by linked drug resistance genes: Neo (neomycin resistance) for Hprt-5BoxB, Hyg (hygromycin B resistance) for λN22HA-DDX6, and Puro (puromycin resistance) for shRNA constructs. (**B**) Methylene blue staining confirmed altered drug resistance and sensitivity in DDX3X-shRNA-expressing mESCs. (**C**) In the λN22HA-DDX6 tethering reporter cell line (1D1), knockdown of DDX3X using two independent shRNAs led to de-repression of Rn-Luc activity without significant increases in mRNA abundance. Shown are relative Rn/Ff-Luc activity ratios from GFP shRNA and DDX3X shRNA-expressing 1D1 cells. (**D**) Similar to panel C, DDX3X was also required for λN22HA-Ago2-mediated translational repression of the Rn-Luc reporter in the previously described 1A2E8 cell line. Results in panels (C) and (D) are presented as mean ± SEM. *P<.05, **P <.01, ***P <.001.

Building on our previously established drug-selection strategy [43], we developed an RNAi-based genetic screen to identify components acting downstream of DDX6. We selected hypoxanthine phosphoribosyl transferase (Hprt) as the reporter gene because it supports both positive selection (with HAT) and negative selection (with 6-TG) (Fig. 2A). To begin, we generated an mESC line expressing Hprt with five copies of BoxB in its 3′ UTR [7–22]. We then introduced a λN22HA-DDX6 transgene to repress Hprt expression, making the reporter cells sensitive to HAT and resistant to 6-TG (referred to as 1G1 cells). Using the 1G1 reporter cells, we carried out a genetic screen with subpools from the mouse retroviral pSM2-shRNA-mir30 library. In total, approximately 40 000 independent puromycin-resistant mESC colonies were screened. We selected 96 HAT-resistant clones for expansion and genomic DNA isolation, subsequently identifying 43 unique candidate targets (Supplementary Table S1). As expected, DDX6 was recovered as a positive control; however, other known DDX6 effectors such as 4E-T were not identified. We also confirmed PTEN’s role in DDX6-mediated translational repression using an independent PTEN shRNA. Notably, this screen identified an shRNA targeting the mouse DDX3X gene. Like DDX6, DDX3X is a member of the DEAD-box helicase family and has been implicated in translational regulation [61]. Further validation using independent shRNAs against DDX3X confirmed that its knockdown in 1G1 reporter cells conferred the expected drug resistance and sensitivity phenotypes (Fig. 2B), indicating that DDX3X is required for DDX6-mediated repression of the *Hprt* reporter gene.

To validate the functional relevance of this novel genetic interaction in translational repression, we demonstrated that knockdown of DDX3X using two independent shRNAs impaired λN22-DDX6-mediated repression of Renilla luciferase in mESCs (Fig. 2C). Furthermore, using the previously described 1A2E8 reporter cell line [43], in which Renilla luciferase is repressed via tethering of λN22-Ago2 to BoxB sites in the 3′UTR, we found that DDX3X knockdown similarly disrupted Ago2-mediated translational repression (Fig. 2D). In both scenarios, Rn-Luc mRNA abundance was not significantly increased (Fig. 2C and D). These findings indicate that DDX3X is required for both DDX6- and Ago2-dependent repression of translation.

The mouse DDX3X gene is located on the X chromosome, while its paralog DDX3Y is expressed at much lower levels in male mESCs. To assess whether DDX3Y contributes to or compensates for DDX3X in translational regulation, we expressed two distinct DDX3Y-specific shRNAs in 1D1 λN22-DDX6 reporter cells. Neither individual nor combined knockdown of DDX3Y with DDX3X significantly affected reporter activity compared to controls (Supplementary Fig. S4). Thus, DDX3Y does not functionally compensate for DDX3X in DDX6-mediated translational repression.

### DDX3X is required for miRNA silencing

To further investigate the role of DDX3X in miRNA silencing, we generated DDX3X cKO ESCs (DDX3X^flox^; CAG-ER^T2^-Cre-ER^T2^) in which exons 5–7 of the endogenous DDX3X gene were deleted upon 4-OHT treatment (Supplementary Fig. S5A). Efficient depletion of DDX3X protein following 4-OHT induction was confirmed by western blot (Supplementary Fig. S5B). Transient puromycin incorporation assays indicated that global protein synthesis rates were not significantly affected in DDX3X-deleted cells (Supplementary Fig. S5C). Consistent with previous reports, both endogenous and Flag-tagged DDX3X were broadly distributed throughout the cytoplasm in mESCs (Supplementary Fig. S5D).

**Figure 3.**
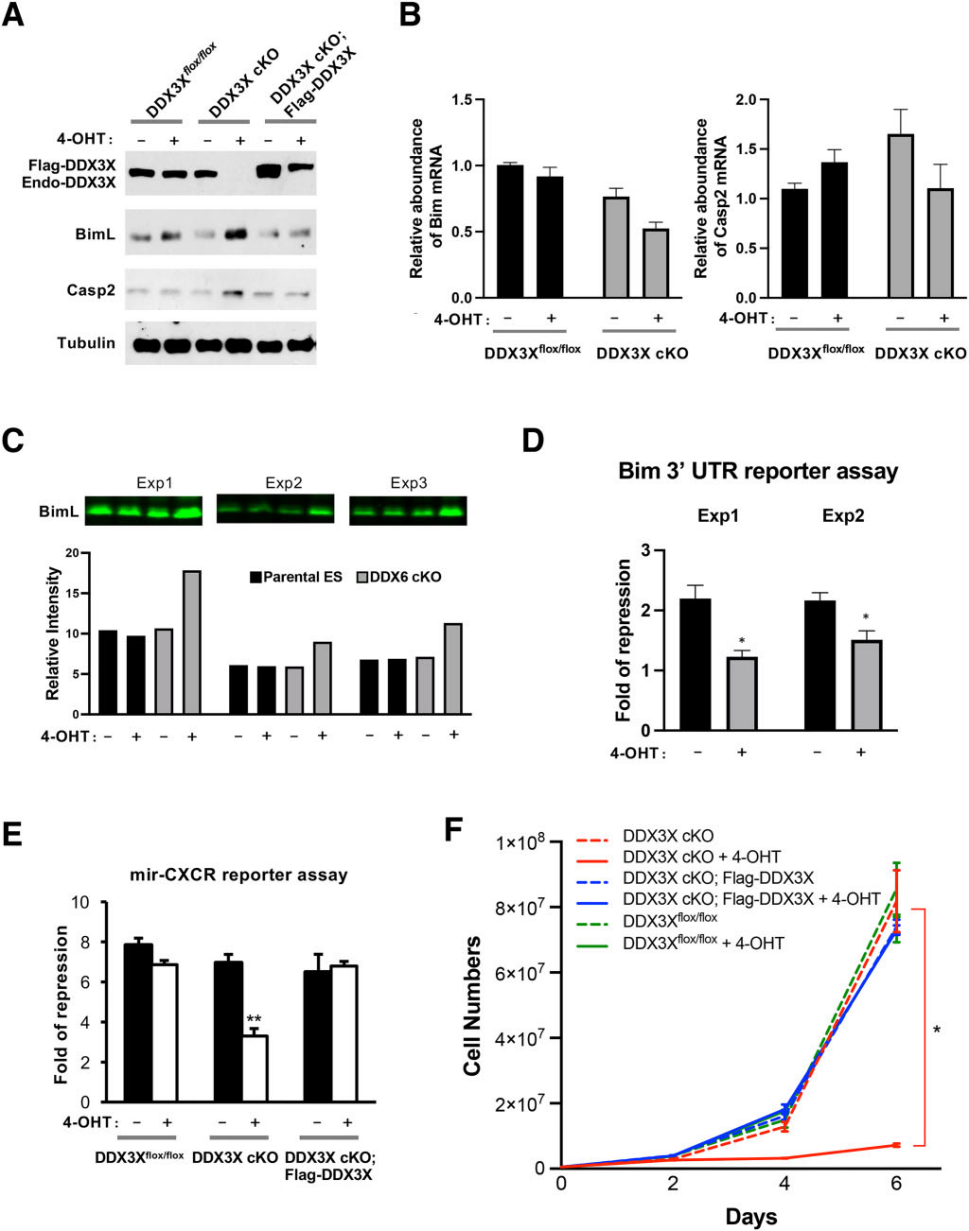
miRNA silencing defects in DDX3X cKO mESCs. (**A**) Endogenous miRNA target proteins Bim and Casp2 were upregulated 2 days after 4-OHT induction in DDX3X cKO mESCs. This defect was rescued by expression of wild-type Flag-tagged DDX3X. (**B**) RT-qPCR analysis showed no corresponding upregulation of Bim and Casp2 mRNAs in DDX3X cKO cells. (**C**) Quantitative western blot analysis confirmed increased Bim protein levels in DDX3X cKO mESCs across three independent experiments. Protein loading was normalized using Revert 700 total protein stain. (**D**) The mir-CXCR reporter assay demonstrated impaired miRNA silencing in DDX3X cKO mESCs, which was rescued by reintroduction of wild-type Flag-DDX3X. (**E**) Similar to DDX6 cKO cells (Supplementary Fig. S2B), DDX3X cKO mESCs treated with 4-OHT were defective in miRNA-mediated Bim 3′UTR reporter silencing. (**F**) DDX3X-deficient mESCs exhibited a growth defect following 4-OHT treatment, which was rescued by wild-type Flag-DDX3X expression. All results are presented as mean ± SEM. *P<.05, **P<.01, ***P<.001.

Supporting the hypothesis that DDX3X functions downstream of DDX6 in the miRNA silencing pathway, we observed increased protein levels of the endogenous miRNA targets Bim and Casp2 following DDX3X depletion (Fig. 3A and C). Consistent with observations in DDX6 cKO cells, Bim and Casp2 mRNA levels were not significantly altered in DDX3X-deficient cells (Fig. 3B), indicating that the observed increase in protein expression results from enhanced translation due to defective miRNA-mediated repression. A Bim-3′UTR reporter assay further demonstrated that endogenous miRNAs repress Rn-luc reporter expression in a DDX3X-dependent manner (Fig. 3D). In addition, mir-CXCR reporter assays confirmed impaired exogenous miRNA silencing in DDX3X-deficient cells (Fig. 3E). Reintroduction of wild-type Flag-tagged DDX3X rescued both the elevated protein expression and reporter repression defects (Fig. 3A and E). Similar to DDX6-null cells, DDX3X knockout ESCs exhibited severe proliferation defect (Fig. 3F), a characteristic phenotype associated with loss of miRNA silencing in pluripotent stem cells [62]. Together, these results establish DDX3X as a critical component in the miRNA silencing pathway, acting downstream of DDX6 to mediate translational repression.

### DDX6 directly interacts with DDX3X

**Figure 4.**
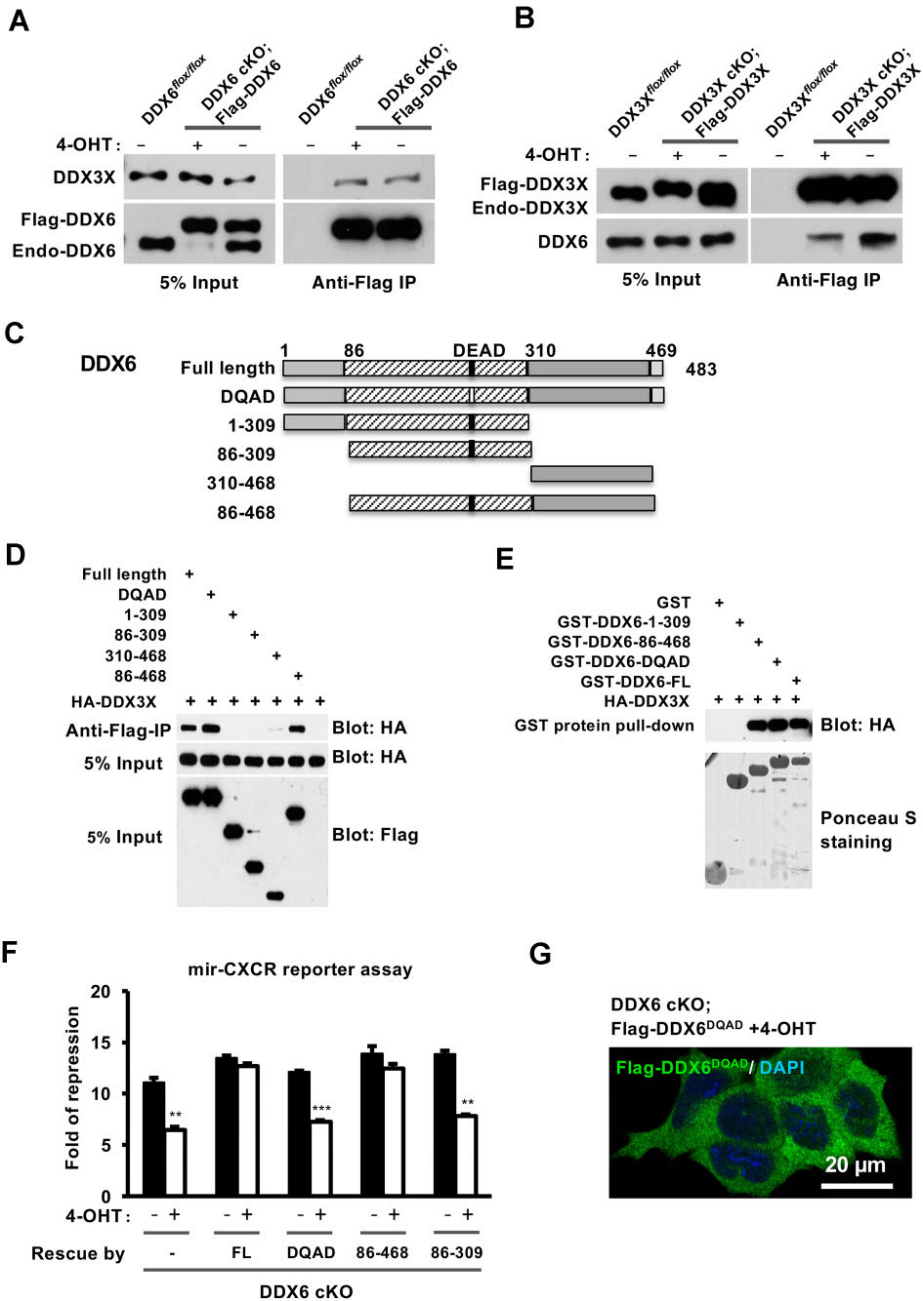
DDX3X interacts with DDX6. (**A, B**) Flag-tagged DDX6 (**A**) and DDX3X (**B**) were expressed at endogenous levels in DDX6 cKO and DDX3X cKO mESCs, respectively. Anti-Flag co-IP demonstrated that DDX6 physically associated with DDX3X in mESCs. (**C**) Schematic diagrams of DDX6 truncation mutants. (**D, E**) Co-IP (**D**) and GST pulldown (**E**) assays using HA-DDX3X-transfected 293T cells identified the DDX6 fragments that interact with DDX3X, revealing that the C-terminal region of DDX6 is required for this association. (**F**) mir-CXCR assay showing that the DDX6^86–468^ variant rescued the miRNA silencing defect in DDX6 cKO mESCs, whereas the DDX6^86–309^ variant, which is defective in DDX3X binding, failed to do so. All results are shown as means ± SEM. *P <.05, **P <.01, ***P<.001. (**G**) The helicase mutant DDX6^DQAD^ was mislocalized throughout the cytoplasm in mESCs.

Our data thus far demonstrate that DDX3X genetically interacts with DDX6 in miRNA-mediated translational repression, prompting the question of whether these proteins also physically associate. To test this, we used genetically rescued cKO mESCs in which Flag-tagged DDX6 or Flag-tagged DDX3X was expressed at levels comparable to their endogenous counterparts. Co-IP with anti-Flag antibodies confirmed that DDX6 and DDX3X interact in mESCs (Fig. 4A and B).

To validate this interaction, we generated a series of DDX6 truncation mutants (Fig. 4C). Co-IP experiments in transfected 293T cells showed that the RecA1-RecA2 domain (amino acids 86–468) was sufficient for binding DDX3X (Fig. 4D). This interaction was independently confirmed by GST pulldown assays using bacterially expressed GST-DDX6 recombinant proteins, which showed efficient binding of DDX3X to the same DDX6 domain (Fig. 4E). Consistent with these findings, expression of Flag-DDX6 (86–468) in DDX6 cKO mESCs rescued the miRNA silencing defect, whereas other truncation mutants failed to do so (Fig. 4F). Notably, a helicase-deficient mutant, DDX6^DQAD^, was unable to rescue miRNA silencing despite retaining its ability to bind DDX3X, likely due to its previously reported mislocalization and turnover defect (Fig. 4G) [63].

**Figure 5.**
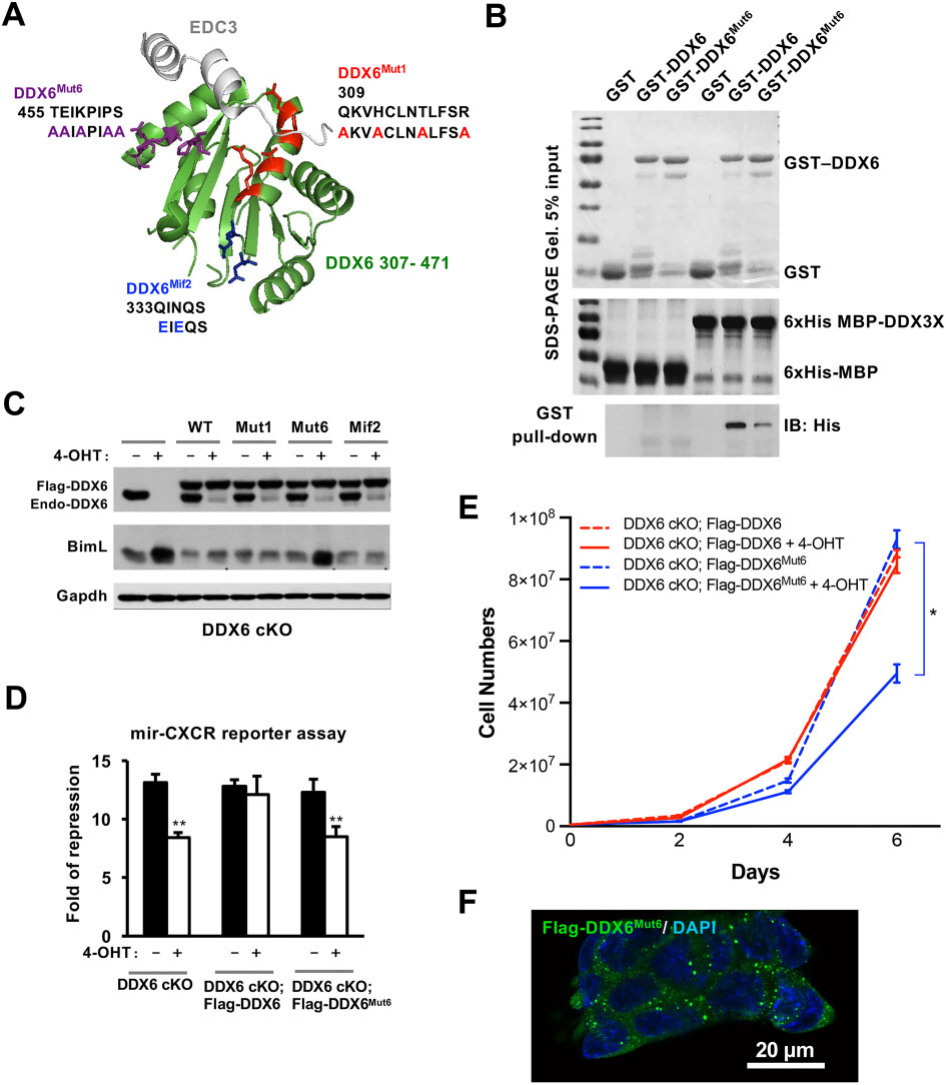
(**A**) DDX3X-interaction mutation in DDX6 impairs miRNA silencing in mESCs. (**A**) Structure of the human DDX6 C-terminal domain in complex with an EDC3 peptide (PDB: 2WAX). Amino acid substitutions in DDX6^Mut1^, DDX6^Mif2^, and DDX6^Mut6^ are highlighted in red, blue, and purple, respectively. (**B**) Direct binding assay of GST-DDX6 and GST-DDX6^Mut6^ to His-MBP-DDX3X^166-590^. DDX6^Mut6^ exhibited reduced binding to DDX3X^166-590^*in vitro*. (**C**) DDX6^Mut6^ failed to rescue the Bim silencing defect, whereas DDX6^Mut1^ and DDX6^Mif2^ successfully did. (**D**) DDX6^Mut6^ also did not rescue the miRNA silencing defect in the mir-CXCR assay. (**E**) DDX6^Mut6^-rescued DDX6 cKO mESCs continued to exhibit a proliferation defect. (**F**) Confocal imaging showed that DDX6^Mut6^ protein localized to P-bodies despite their defect in translational silencing. All results are presented as means ± SEM. *P<.05, **P<.01, ***P <.001.

To define the regions of DDX6 required for interaction with DDX3X, we generated a panel of smaller C-terminal truncation mutants (ΔC10–ΔC110). Co-IP experiments demonstrated a progressive loss of DDX3X binding with increasing truncation, suggesting that the C-terminal region of DDX6 is essential for this interaction (Supplementary Fig. S6A). Prior studies have shown that the RecA2-C region of DDX6 is also important for interactions with other miRISC components [29, 38, 39, 42], including EDC3, 4E-T, and CNOT1. The binding interfaces for these partners have been previously mapped (Fig. 5A). For instance, alanine substitution of residues in Mut1 (red) [42] disrupts DDX6’s interaction with Edc3, 4E-T, and Pat1l, while glutamic acid substitution in Mif2 (blue) [39] impairs its interaction with CNOT1 (Fig. 5A).

To further investigate the molecular interface between DDX6 and DDX3X, we designed a new DDX6 mutant (designated DDX6^Mut6^) by introducing alanine substitutions (purple) on the surface of DDX6 opposite to the previously characterized DDX6^Mut1^ and DDX6^Mif2^ interaction sites (Fig. 5A). We expressed and purified both wild-type DDX6 and DDX6^Mut6^ as GST-tagged recombinant proteins from *E. coli*, along with His-MBP-tagged DDX3X^166-590^, which encodes the full helicase domain. *In vitro* binding assays using these purified proteins showed that wild-type DDX6 directly interacts with DDX3X, whereas the DDX6^Mut6^ mutation significantly impaired this interaction (Fig. 5B). Additionally, we measured the *in vitro* binding kinetics between purified His-MBP-DDX3X and GST-DDX6 proteins using a BLItz assay. The estimated equilibrium dissociation constants (*K*_D_) were 389 nM for DDX6 and 9.48 mM for DDX6^Mut6^, respectively. Thus, these results identify the DDX6^Mut6^ surface as a critical interface for DDX3X binding. In conclusion, our data demonstrate that DDX6 directly interacts with DDX3X both *in vivo* and *in vitro*, providing a biochemical basis for their functional interaction in miRNA-mediated translational repression.

### miRNA silencing defects of DDX6^Mut6^-expressing mESCs

Our biochemical characterization of the DDX6–DDX3X interaction identified a C-terminal surface of DDX6 as the interface responsible for DDX3X recognition. To assess whether mutation of this interface affects DDX6’s interactions with other known partners, we performed co-IP experiments to compare the complex composition of wild-type and mutant DDX6 proteins (Supplementary Fig. S6B). Relative to wild-type DDX6, DDX6^Mut6^ showed reduced binding to DDX3X, while maintaining similar binding to EDC3 and exhibiting increased association with CNOT1. As expected, DDX6^Mut1^ and DDX6^Mif2^ displayed impaired binding to their respective known interactors (EDC3/4E-T and CNOT1, respectively) without affecting DDX3X binding (Supplementary Fig. S6B). Despite these binding defects, mESCs expressing DDX6^Mut1^ or DDX6^Mif2^ showed only modest or no impairment in mir-CXCR reporter assays (Supplementary Fig. S6C).

To determine the functional significance of the DDX6-DDX3X interaction, we stably transfected DDX6 cKO mESCs with a PiggyBac transgene expressing Flag-DDX6^Mut6^. Following deletion of endogenous DDX6, DDX6^Mut6^ failed to repress endogenous Bim protein expression (Fig. 5C). Consistently, miR-CXCR reporter assays revealed a silencing defect for exogenous miRNA in these cells (Fig. 5D). In line with these persistent silencing defects, DDX6^Mut6^-rescued mESCs exhibited continued proliferation defects (Fig. 5E), despite the mutant protein displaying normal subcellular localization (Fig. 5F). Together, these data support the conclusion that the direct interaction between DDX6 and DDX3X is crucial for miRNA silencing.

### Inhibition of *in vitro* translation by DDX6

To explore how DDX6 functions through DDX3X, we examined the possibility that DDX3X acts either as a translation repressor, like DDX6, or as a component of the translation machinery. We tested the repressor activity of DDX3X using the λN22 tethering system in 1A2 reporter mESCs (Supplementary Figs S3 and S7A). Unlike λN22-DDX6, tethered λN22-DDX3X failed to efficiently suppress translation of the BoxB-containing Rn-Luc mRNA, arguing against a direct repressor function for DDX3X.

**Figure 6.**
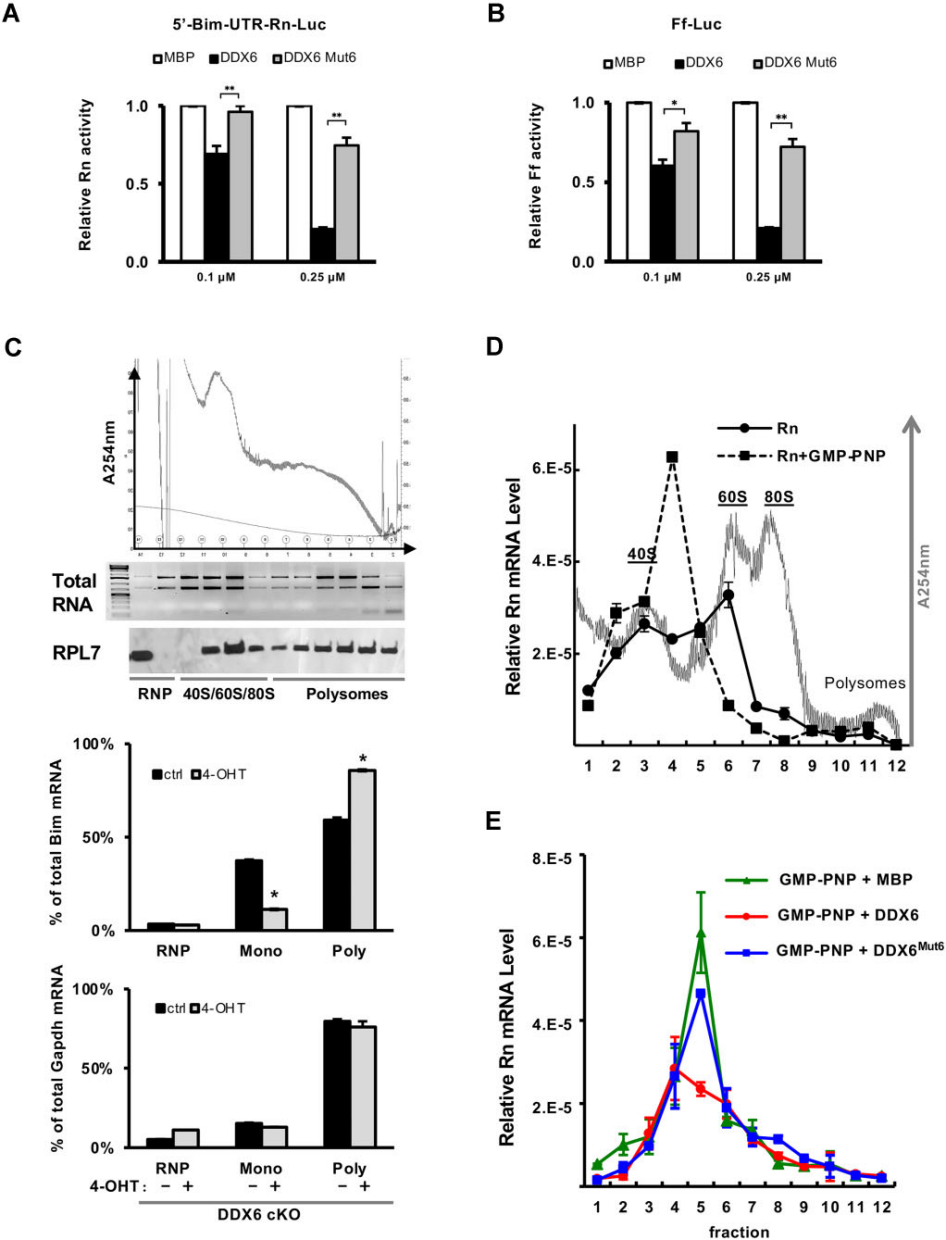
Inhibition of translation by DDX6. (**A**, **B**) His-MBP-DDX6 inhibited the *in vitro* translation of both 5′Bim-UTR-Rn-Luc-pA (**A**) and Ff-Luc-pA (**B**) mRNAs, whereas His-MBP-DDX6^Mut6^ did not. (**C**) Sucrose gradient ultracentrifugation analysis of monosomes and polysomes in DDX6 cKO mESCs. Ultraviolet absorbance at 254 nm (A254nm), ribosomal RNA (rRNA), and the ribosomal protein RPL7 were used to identify ribonucleoprotein (RNP), monosome, and polysome fractions. Bim and GAPDH mRNA levels in these fractions were analyzed by RT-qPCR. (**D**) Sucrose gradient ultracentrifugation analysis showed that addition of GMP-PNP inhibited translation initiation, resulting in accumulation of the 48S PIC (**E**) In the presence GMP-PNP, MBP-DDX6 blocked 48S PIC formation, whereas MBP-DDX6^Mut6^ or MBP alone did not. All results are shown as means ± SEM. *P <.05, **P<.01, ***P<.001.

We next evaluated whether DDX6 regulates translation via its interaction with DDX3X using an *in vitro* translation system derived from rabbit reticulocyte lysates. DDX3X has been previously reported to interact with multiple components of the translation machinery [61], and, as expected, we confirmed its abundant presence in commercial reticulocyte lysates (Supplementary Fig. S7B). *In vitro* translation was performed using two poly(A)-containing mRNA substrates (Fig. 6A and B). For Rn-Luc mRNA, a 235-nt 5′UTR from the mouse *Bim* gene was placed upstream of the Rn-Luc coding sequence (Fig. 6A). In both cases, wild-type MBP-DDX6 repressed luciferase protein synthesis in a dose-dependent manner, whereas the DDX3X-binding mutant MBP-DDX6^Mut6^ exhibited markedly reduced activity. These results suggest that DDX6-mediated translational repression depends on its interaction with DDX3X.

A previous study demonstrated that DDX3X interacts with the eIF3 complex [64]. To assess whether DDX6 disrupts with this interaction, we performed a competitive binding assay using affinity-purified Flag-DDX3X as bait (Supplementary Fig. S7C, D). In this assay, representative eIF3 subunits eIF3A and eIF3F were efficiently co-purified with Flag-DDX3X. However, the addition of recombinant wild-type MBP-DDX6 competitively inhibited the DDX3X–eIF3 interaction, whereas MBP-DDX6^Mut6^ had no such effect (Supplementary Fig. S7D), suggesting that the DDX6-DDX3X interaction may interfere with translation initiation.

Translation initiation is a key step in miRISC-mediated translational regulation [14]. The yeast DDX6 ortholog Dhh1p is known to inhibit translation initiation [40]. To investigate whether DDX6 functions similarly, we performed sucrose gradient ultracentrifugation to examine mRNA distribution in DDX6 cKO mESCs. Sucrose fractions were pooled into three groups: low-density sRNP, medium-density monosomes (Mono), and high-density polysomes (Poly). RT-qPCR analysis of Bim and GAPDH mRNAs across these fractions revealed that, following DDX6 depletion by 4-OHT treatment, a substantial portion of miRNA-targeted Bim mRNA shifted from monosome to polysome fractions (Fig. 6C), indicating increased translation. In contrast, the distribution of nonmiRNA-targeted GAPDH mRNA was unaffected by DDX6 deletion (Fig. 6C). These findings demonstrate that DDX6 represses Bim mRNA translation, promoting its accumulation in the monosome fractions under normal conditions.

To further define the step at which DDX6 inhibits translation initiation, we performed sucrose gradient ultracentrifugation of *in vitro* translation reactions using rabbit reticulocyte lysate. In the presence of the nonhydrolyzable GTP analog GMP-PNP, translation initiation is stalled at the stage of 48S PIC formation [65] (Fig. 6D). To compare the inhibitory effects of wild-type DDX6 and DDX6^Mut6^, we conducted this assay and found that wild-type DDX6 efficiently inhibited 48S PIC formation, consistent with the activity of the yeast ortholog Dhh1p [40]. In contrast, DDX6^Mut6^ failed to inhibit PIC formation *in vitro* (Fig. 6E). These findings suggest that DDX6 interferes with a step leading to PIC assembly, likely through its interaction with DDX3X.

## Discussion

DDX6 plays a pivotal role in post-transcriptional regulation, including P-body assembly, mRNA decapping, translational repression, and codon optimality-mediated mRNA degradation [28, 40, 63, 66–70]. Despite extensive research, the molecular mechanisms underlying DDX6-mediates translational repression have remained elusive. It has long been hypothesized that DDX6 inhibits translation initiation by interacting with unknown binding partners [11, 15]. In this study, we identify DDX3X as the long-sought binding partner of DDX6 in this process and demonstrate that these two DEAD-box RNA helicases interact to repress translation at the initiation stage.

**Figure 7.**
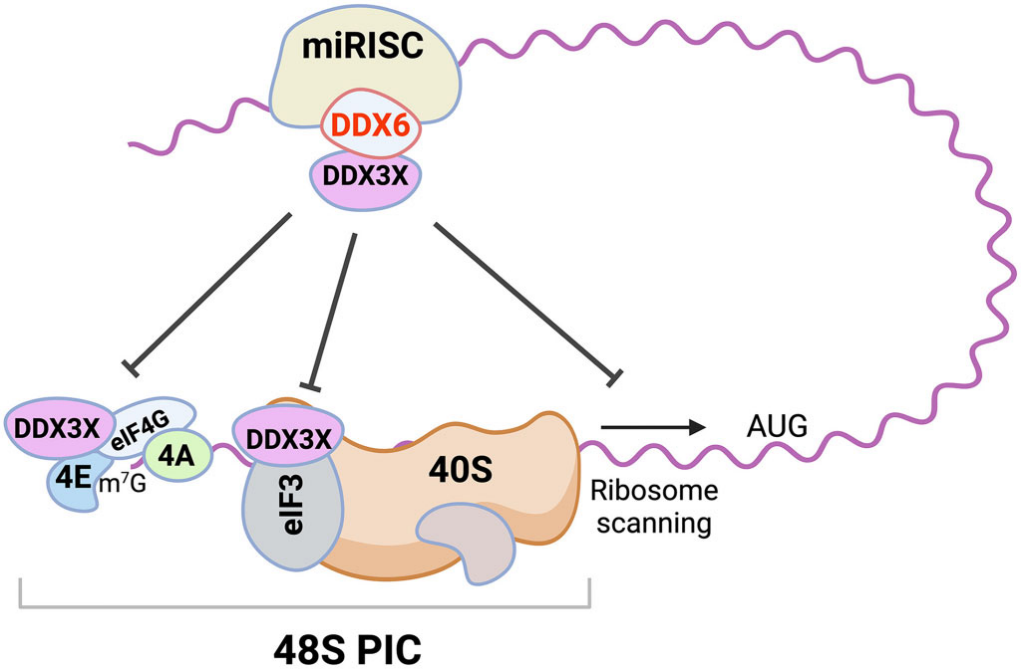
Proposed model of DDX6-mediated translational repression in miRNA silencing. DDX3X is involved in multiple steps of translation initiation. The interaction between DDX6 and DDX3X may disrupt one or more of these steps, thereby inhibiting the formation of the 48S PIC.

Similar to DDX6, DDX3X has recently emerged as a key regulator of translation [71]. DDX3X interacts with various components of the translation machinery, including ribosomal proteins, 18S rRNA, and multiple eukaryotic initiation factors such as eIF3, eIF4A, eIF4E, and eIF4G [61]. Global CLIP-seq analyses further reveal that DDX3X associates with nearly all mRNAs, with a binding preference at the 5′UTR [72–76]. Its presence in the eIF4F complex and the PIC underscores its central role in regulating translation initiation [61]. We propose that DDX6 exerts its inhibitory effect on translation by engaging with DDX3X within one or more of these complexes. The key question is how DDX6 and DDX3X cooperate to mediate translational repression. As a working model (Fig. 7), several potential mechanisms are proposed and discussed below.

In one scenario, DDX6 may interfere with the formation of the eIF4F complex through its interaction with DDX3X. In yeast, the DDX3X ortholog Ded1 has been shown to interact with eIF4E, eIF4G, and eIF4A [74, 77–80]. These interactions facilitate 48S PIC assembly during translation initiation [81]. However, it remains unclear whether mammalian DDX3X functions in a similar manner. DDX3X and Ded1 differ in their N- and C-terminal sequences, which are responsible for the direct interactions between Ded1 and eIF4F components. Although a putative eIF4E-binding motif (YIPPHLR) exists in the N-terminus of DDX3X [82], other studies suggest this motif is dispensable for its function in translation [83, 84]. Nevertheless, DDX3X appears to associate with the eIF4F complex [73, 83–85], and its interaction with DDX6 may disrupt eIF4F assembly, thereby inhibiting translation initiation.

Alternatively, scanning of the 43S PIC has been proposed as a key step through which miRISC represses translation [21, 86]. eIF4A was initially considered a primary candidate for this role [86–88]. However, recent studies have argued against the notion that eIF4A is essential for this process [14, 15, 89]. The identification of DDX3X as a downstream target for DDX6 in this study offers an alternative explanation. DDX3X and eIF4A may have overlapping or redundant functions during 43S PIC scanning to promote the assembly of eIF4F and 43S PIC into the 48S PIC [90]. While the role of mammalian DDX3X in scanning remains less defined, studies in yeast strongly support a function for its ortholog Ded1 in this step [81, 91]. Thus, DDX6 may inhibit translation initiation at this stage by blocking DDX3X.

Finally, eIF3 orchestrates the formation of both the 43S and 48S PICs [92]. Recent cryo-EM structures of the 48S PIC highlight the essential contributions of eIF3 subunits to translation initiation complex assembly [93]. DDX3X has been known to interact with the eIF3 complex [64]. As shown in Supplementary Fig. S7, recombinant DDX6 competitively inhibits the interaction between DDX3X and eIF3, suggesting that DDX6 may sequester DDX3X away from eIF3. This interference may prevent DDX3X from engaging with other essential PIC assembly factors. For example, eIF4A has been shown to modulate eIF3j binding to the ribosomal subunit to facilitate mRNA access to the entry channel of the 43S PIC [94]. By analogy, DDX3X and eIF4A may share similar functions, with DDX6 counteracting DDX3X activity in this context. Supporting this model, both Ded1 and DDX3X bind to helix 16 of the small ribosomal subunit [72, 74], in close proximity to the mRNA entry channel.

Considering these potential mechanisms by which DDX6 targets the translation initiation machinery, a critical next step toward understanding this regulatory process is to elucidate the structural basis of DDX3X interactions with its associated complexes—including DDX6, eIF4F, the 43S PIC, and the 48S PIC. Such insights would enable the rational design of separation-of-function variants and facilitate a mechanistic dissection of DDX6 activity at distinct stages of translation initiation *in vivo*.

## Supplementary Material

gkaf868_Supplemental_File

## Data Availability

The data underlying this article are available in the article and in its online supplementary material. Reagents including expression vectors and shRNAs described in this work will be deposited and distributed through Addgene.
