# Supplementary material for: DDX6 interacts with DDX3X to repress translation in microRNA-mediated silencing
Source: Nucleic Acids Res. 2025 Sep 9;53(17):gkaf868. doi: 10.1093/nar/gkaf868 (PMC12418388; doi:10.1093/nar/gkaf868)
Supplement: gkaf868_Supplemental_File [file gkaf868_supplemental_file.pdf]

Supplementary Materials to

DDX6 interacts with DDX3X to repress translation in microRNA-mediated silencing

Yanyan Lu,[1] Meng Tao,[1] Hong Su,[1] Yiren Tu,[1] Ji-Ping Wang,[2] Masahiko Kuroda, [3] and Xiaozhong Wang[1, *]

[1]Department of Molecular Biosciences, Northwestern University, Evanston, IL 60208, USA

[2]Department of Statistics and Data Science, Northwestern University, Evanston, IL 60208, USA

[3]Department of Molecular Pathology, Tokyo Medical University, 6-1-1, Shinjuku, Shinjuku-ku, Tokyo, 160-8402, Japan.

[*]Correspondence: awang@northwestern.edu (X.W.)

Table S1: Candidate RNAi targets identified by shRNA sequencing

	Gene	Full Name
1	Ppef2	Protein phosphatase with EF-hand domain 2
2	Tsg101	Tumor susceptibility 101
3	Cndp1	Carnosine dipeptidase 1
4	Olfr135	Olfactory receptor family 2 subfamily N member 1C
5	Morn1	MORN repeat containing 1
6	Shc1	Src homology 2 domain-containing transforming protein C1
7	Fkbp4	FK506 binding protein 4
8	Hsp90	Heat shock protein 90
9	Pten	Phosphatase and tensin homolog
10	Smarca5	SNF2 related chromatin remodeling ATPase 5
11		RIKEN cDNA 5830472M02
12	CD22	B-cell receptor CD22
13	Xpo6	Exportin 6
14	Mmp20	Matrix metalloproteinase 20
15	Lrrc2	Leucine-rich repeat-containing protein 2
16		RIKEN cDNA 4921506M07
17		RIKEN cDNA D230037D09
18	Col23a1	Procollagen, type XXIII, alpha 1
19	Pdcd1	Programmed cell death 1
20	Taar4	Trace amine-associated receptor 4
21	Apaf1	Apoptotic protease activating factor 1
22	Kcnab1	Potassium voltage-gated channel, shaker-related subfamily, beta member 1
23	Slc24a3	Sodium/potassium/calcium exchanger 3
24	Hk2	Hexokinase 2
25	DDX3X	DEAD-box helicase 3 X-linked
26	Syt14	Synaptotagmin XIV
27	Ankrd15	KN motif and ankyrin repeat domain-containing protein 1
28	Zfp184	Zinc finger protein 184
29	Apob	Apolipoprotein B
30	DDX6	DEAD-box helicase 6
31	Dusp18	Dual specificity phosphatase 18
32	GPR74	G protein-coupled receptor 74
33	Tnfrsf19	Tumor necrosis factor receptor superfamily, member 19
34	Olfr1044	Olfactory receptor 1044
35	Tnfaip6	Tumor necrosis factor alpha induced protein 6
36	Impa1	Inositol (myo)-1(or 4)-monophosphatase 1
37	TAF9	TATA box binding protein (TBP)-associated factor 9
38	Rab11fip2	RAB11 family interacting protein 2
39	MRLP15	Mitochondrial ribosomal protein L15
40		Hypothetical protein 9630025C22
41	Krtap8-2	Keratin associated protein 8-2
42	Pygl	Liver glycogen phosphorylase
43		RIKEN cDNA 4921529O18

Table S2: Primers used in this paper

**DDX6 targeting screen primers**	
	3305. DDX6-3PRB-S: GGTGACCAAGGGAACATTAAATAATAGCTAGAG
	3306.DDX6-3PRB-AS: ACACATTCAAGGTTCTTTTACAGTTAGCGTGC
	3307.DDX6-5PRB-S: AGACATCACTTCATGTAAACAGGACTGGCCTC
	3308.DDX6-5PRB-AS: AGGGAGTTTCCAGTAGACTACCTAGTAACCAG
	3309.DDX6-LOXP-S: GTTCATCCTTTTTGTTATATGAAGGTTATGTATCATGC
	3310.DDX6-LOXP-AS: CAAGGACACGTTACCATGTTACACTATGGCTC
	3232-LAR3-AS: CCACAACGGGTTCTTCTGTTAGTCC
	3233-RAF5-S: CACACCTCCCCCTGAACCTGAAAC
	3234.PNF: ATCCGGGGGTACCGCGTCGAG
	3235.R2R: TTGATATCTCTATAGTCGCAGTAGGCG
	3236.D6GF3: CCTTACGGTTACCGGACTCCGATTC
	3237.D6GF4: CTCTGGACGAGCGGTGCGGTAGAC
	3238.D6GR3: GACCAGTAAGACAAGCATAGCCTAGCGTAC
	3239.D6GR4: CAATGACCAGTAAGACAAGCATAGCCTAG
**DDX6 cDNA cloning and site-specific mutagenesis**	
	1366.DDX6.MFE.ATG.S: GTCATACAATTGCTATGAGCACGGCCAGAACAGAGAA
	1367.DDX6.XHOI.AS: GCAAACTCGAGATGTGTCACAGATCCAAAGAGC
	3201.DDX6.R1.S: GAACGAATTCTGAAAGGAGTAACTCAGTACTACG
	3202.DDX6.STOP.XHO.AS: TACTCCTCGAGTTACAGAGTTAGTTCCTCCATCAGG
	3218: DDX6N.MFE: GATCTAACAATTGTTAAAACTTCGGATGTGACCTCCACA
	3222-DDX6-DQAD-AS: TCCTGTGACAACAATTTATCTGCCTCATCTAGCAC
	3223-DDX6-DQAD-S: TGTCCAGATGATAGTGCTAGATGAGGCAGATAAATTGTTG
	3557-DDX6-MUT1-S: AGTGGCCTGCCTCAATGCACTTTTCTCCGCGCTTCAGATAAACCAGTCCATC
	3558-DDX6-MUT1-AS: GAGAAAAGTGCATTGAGGCAGGCCACTTTTGCGCGCTCCGTTACATATGCGTAG
	3794-DDX6-DEL-MUT6-AS: TTGATCTCGAGTCACAGCTGCTCCTCAATACTCTTCAGGTTG
	3795-DDX6-DELC20-AS: ACAGGCTCGAGTCAGATGTTGCTTGGGATGGGTTTGATCTC
	3717-DDX6-MUT6-S: GCAGCGATCGCACCCATCGCAGCAAACATCGACAAGAGCCTGTATGTGGCAG
	3718-DDX6-MUT6-AS: AACTTGATCGCTGCTGCTGATGCGTTCAACCTGAAGAGTATTGAGGAGCAG
	3721.DDX6-DELC10-AS: GGCTCACTCGAGTTATTCTGCCACATACAGGCTCTTG

	3722.DDX6-STOP-XHOI-AS: AAGCGCTCGAGTTACGGTTTCTCGTCTTCTGCAGGC
	4172-DDX6-MIF2-S: ACACTTTTCTCCAGGCTTGAGATAGAGCAGTCCATCATTTTCTGCAACTCC
	4173-DDX6-MIF2-AS: GAAAATGATGGACTGCTCTATCTCAAGCCTGGAGAAAAGTGTATTGAGGCAG
**DDX3X targeting screen primers**	
	3330.DDX3X-2LOXP-S: CTATAATCTGAACAATTGATGTTTGGGGGTGC
	3232-KAR3-AS: CCACAACGGGTTCTTCTGTTAGTCC
	3233-RAF5-S: CACACCTCCCCCTGAACCTGAAAC
	3234.PNF: ATCCGGGGGTACCGCGTCGAG
	3235.R2R: TGATATCTCTATAGTCGCAGTAGGCG
	3240.D3GF3: GAGAGGCCTGAGGCAAGTTATCTCGGTCAG
	3241.D3GF4: CGCAGCTTGAACAGAGTAATGTGAATCGGC
	3242-D3GR3: CAGCTTGAACAGAGTAATGTGAATCGGC
	3243.D3GR4: CGCAGCTTGAACAGAGTAATGTGAATCGGC
	3323. DDX3-LOXP-S: CTTGATGGCTTGTGCTCAAACAGGTAAGCTCA
	3324. DDX3-LOXP-AS: TTCTCCTGGACCATCAGCATAGATCTGACTCA
	3325.LOXP-RV-AS: GGTCTGAGCTCGCCATCAGTTCA
	3326.3LOXP-RV-AS1: TGAACTGATGGCGAGCTCAGACCATAACTT
	3327.3LOXP-RV-AS1: CGAAGTTATCATTAATTGCGTTGCGCCATCTC
	3328.2LOXP-S: AAGGCGCATAACGATACCACGATATCAACAAGT
	3329.2LOXP-5235-AS: CCGCCTACTGCGACTATAGAGATATCAACC
**DDX3X cDNA cloning and site-specific mutagenesis**	
	2971-hDDX3X.R1.S: GATTAAGAATTCTGATGAGTCATGTGGCAGTGGAA
	2972-hDDX3X.Xho.AS: TGAAGCTACTCGAGGTATAGTCTGGCTAAG
	3225.hDDX3X.R1.S: GAGTTAGAATTCCAGTTGAGGCAACAGGCAACA
	3178.hDDX3X.XHO.STOP.AS: CTAATACTCGAGTTAACCCTTGTAGTGGTGTTCATAAGCC
**shRNA primers**	
	2967. mDDX3X.shRNA1.S: TTTGCCACCTCATTCTTTAATGAAACTAGAGTTTCATTAAAGAATGAGGTGGTTTTTC
	2968.AS: TCGAGAAAAACCACCTCATTCTTTAATGAAACTCTAGTTTCATTAAAGAATGAGGTGG
	2973. mDDX3X.shRNA2.S: TTTGACGTTCTAAGAGCAGTCGATTCTAGAGAATCGACTGCTCTTAGAACGTTTTTTC
	2974.AS: TCGAGAAAAAACGTTCTAAGAGCAGTCGATTCTCTAGAATCGACTGCTCTTAGAACGT
	3084. mDDX3X.shRNA3.S: TTTGCTGTGATTCTCCACTGAAATCATTAGATTTCAGTGGAGAATCACAGCTTTTTC

	3085.AS: TCGAGAAAAAGCTGTGATTCTCCACTGAAATCTAATGATTTCAGTGGAGAATCACAG
	5782.mDDX3Y.sh1.S: TTTGCCAAGCGATATTGAAGAATACATTAGTATTCTTCAATATCGCTTGGCTTTTTC
	5783.AS: TCGAGAAAAAGCCAAGCGATATTGAAGAATACTAATGTATTCTTCAATATCGCTTGG
	5784.mDDX3Y.UTR.sh2.S: TTTGCTTTGTTAATTCTGGTCTTTCATTAGAAAGACCAGAATTAACAAAGCTTTTTC
	5785.AS: TCGAGAAAAAGCTTTGTTAATTCTGGTCTTTCTAATGAAAGACCAGAATTAACAAAG
**qPCR primers:**	
	2604. BIM.RT.AS: GGCTGCATGTAGATCCTGTCAATG
	2608.BIM.QPCR.S1: GCTTCCATACGACAGTCTCAGGA
	2609.BIM.QPCR.S2: GGAGATACGGATTGCACAGGAG
	2644.FFLUC.QPCR.S: GAGGTTCCATCTGCCAGGTATC
	2645.FFLUC.QPCR.AS: CCGGTATCCAGATCCACAACC
	2646.RLUC.QPCR.S: AACGCGGCCTCTTCTTATTTATGG
	2647.RLUC.QPCR.AS: AGATTTGCCTGATTTGCCCATACCA
	5778.mCasp2.qPCR.S: TTCATCCAAGCATGTCGTGGAG
	5779. mCasp2.qPCR.AS: TGTTCCGCATGGCAGCATTAC
**In vitro transcription primers**	
	3580.T7.RNLUC.S: CAATTCTAATACGACTCACTATAGGGCACCTATTGGTCTTACTGAC
	3581.T30.RNLUC.AS: TTTTTTTTTTTTTTTTTTTTTTTTTTTTTTCTGCATTCTAGTTGTGGTTTGTCC
	3629.BIM5'UTR.UP.S: CTGGCTAACTAGAGAACCCACTG
	3630. RNLUC.PA.AS1: TTTTTTTTTTTTTTTTTTTTTTTTTTTTTTAGCCCCAGCTGGTTCTTTCC
**microRNA:** The oligos were mixed 1:1 and annealed (80°C for 5 minutes, then the temperature was decreased 0.01°C/sec to 50°C and held at 50°C for 30 minutes then decreased at 2°C/sec to 4°C).	
	CXCR miRNA: TOP: 5'-GUU UUC ACA AAG CUA ACA CA-3'
	CXCR miRNA BOTTOM: 5'-UGU UAG CUG GAG UGA AAA CUU-3'
	mir-30 TOP: GCU GUA AAC AUC CUC GAC UGG AAG UU
	mir-30 BOTTOM: CUU UCA GUC GGA UGU UUG CAG CUU

Table S3: Antibodies used in this paper

DDX6	Bethyl A310-131A, rabbit anti-DDX6, 1:5000, MW 55 KDa<br>Bethyl A310-460A, rabbit anti-DDX6, 1:5000
DDX3X	Bethyl A300-474A, rabbit anti-DDX3, 1:5000, MW 75kDa
Flag	Sigma F3165, Anti-Flag M2 Monoclonal Antibody, 1:2000
HA	Sigma H6908, rabbit anti-HA antibody, 1:2000<br>Roche Appplied Science, 11 583 816 001, Mouse, 1:2000
V5	AbD Serotec Clone SV5-PK1 mouse, 1:2000
Myc	DSHB, 9E10 mouse antibody 1:20
GFP	Invitrogen, A-11122, rabbit antibody, 1:1000
Bim	Enzo Life Sciences, ADI-AAP-330, rabbit anti-Bim/BOD polyclonal Ab, 1:1000, MW 23, 16, 13 kDa
Bim	Cell Signaling 2933, rabbit mAb (C34C5), 1;1000, MW 23, 16, 13 kDa
4E-T	Fortis, A300-706A, rabbit anti-4E-T/eIF4E-T antibody affinity purified, 1:1000, 110 KDa
His	Novagen 70796-4, His Tag Monoclonal antibody, 1:4000
GST	Proteintech, 66001-1-Ig, Mouse antibody, 1:50000, MW 26kDa
Caspase 2	Cell Signaling #2224, mouse antibody, 1:1000, MW 12, 14, 48kDa
GAPDH	GAPDH (6C5) antibody Santa Cruz Biotechnology Cat# sc-32233, RRID:AB_627679
Dcp2	Bethyl A302-597A, Rabbit anti-Dcp2, 1:2000, MW about 55kDa
Puromycin	Mouse Anti-Puromycin antibody (3RH11), KeraFAST, 1:2000
eIF3A	Cell Signaling 3411, anti-eIF3A (D51F4) XP TM Rabbit mAb, 1:1000, MW 166 kDa
RPL7	Bethyl, A300-740A, MW 29kDa
Tubulin Beta III isoform	Millipore MAB1637, Mouse, 1:2000, MW 55 kDa
Flag	Anti-FLAG M2 Affinity Gel, Sigma, A2220
Total protein quantification	LI-COR P/N 926-11010, Revert 700 Total protein Stain Kit
HRP-conjugated secondary antibody	Goat anti-Mouse IgG(H/L):HRP, BioRad 5178-2504, 1:100-1:5000<br>Goat anti-rabbit IgG(H/L):HRP, BioRad, 5196-2504 , 1:1000-1:5000
DyLight™ 800 secondary antibody	Invitrogen SA5-35571, goat anti-Rabbit IgG DyLight™ 800

FITC- or Cy3 conjugated secondary antibody	CyTM3-conjugated AffiniPure Donkey Anti-Rabbit IgG (H+L), Jackson ImmunoResearch, 711-165-152, 1:200<br>Alexa Fluor 488-conjugated AffiniPure Goat Anti-Mouse IgG (H+L), Jackson ImmunoResearch 115-545-166, 1:200<br>Alexa Fluor 488-conjugated AffiniPure Donkey Anti-Rabbit IgG (H+L), Jackson ImmunoResearch 711-545-152, 1:200

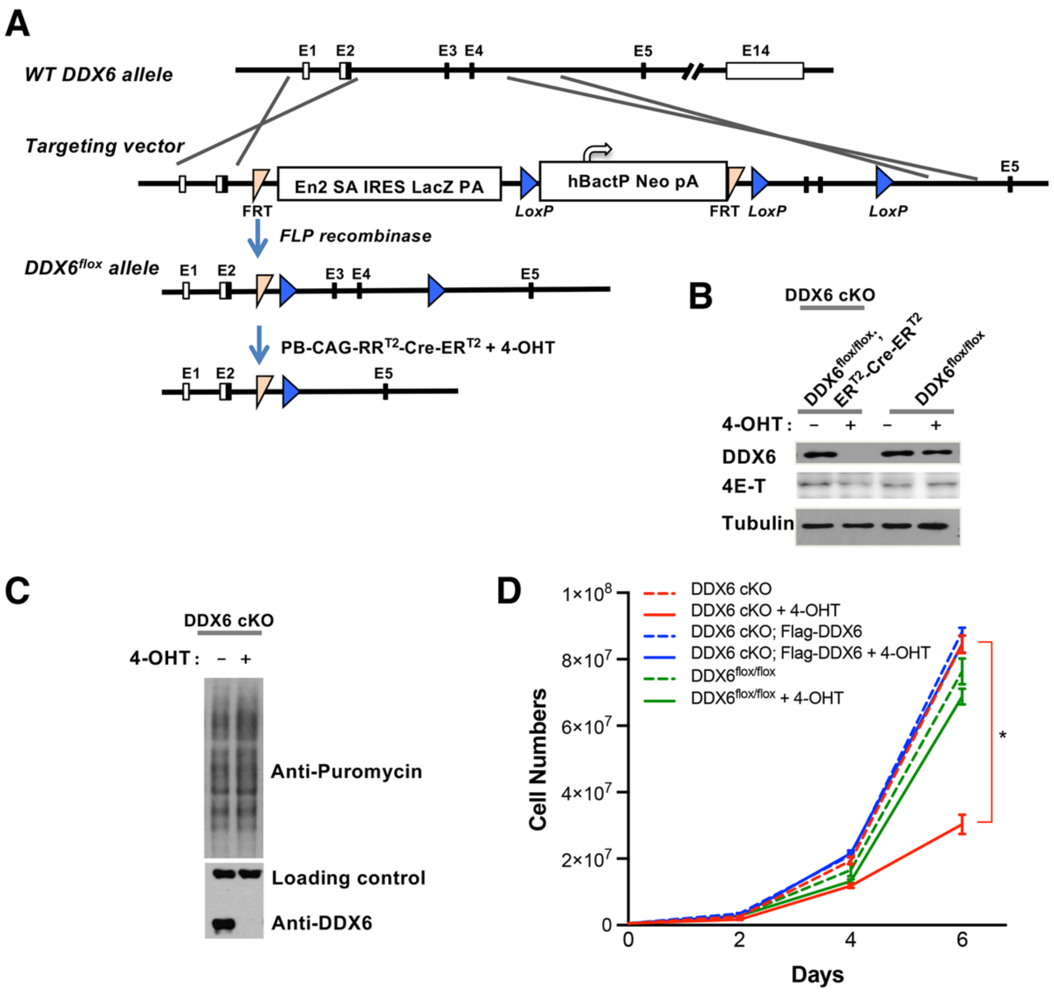

Figure S1. An inducible DDX6 knockout strategy in mESCs. A. Shown are the wild-type locus and targeting vector for the mouse DDX6 gene. Exon 3 and exon 4 are flanked by two loxP sites in the targeted $DDX6^{flox}$ allele. **B.** Upon 4-OHT activation, Cre excised exons 3 and 4, resulting in a complete loss of DDX6 protein, as confirmed by western blot analysis. 4E-T and tubulin served as controls. **C.** Western blot analysis of puromycin pulse-labeled total proteins in DDX6 cKO cells. **D.** Growth curves of DDX6 cKO mESCs with or without 4-OHT treatment. mESCs deficient in DDX6 exhibited a growth defect 4 days after 4-OHT treatment. Expression of a wild-type Flag-DDX6 transgene completely rescued the growth phenotype. Error bars in D indicate mean ± SEM for three cell counts. *$p < 0.05$, **$p < 0.01$, ***$p < 0.001$.

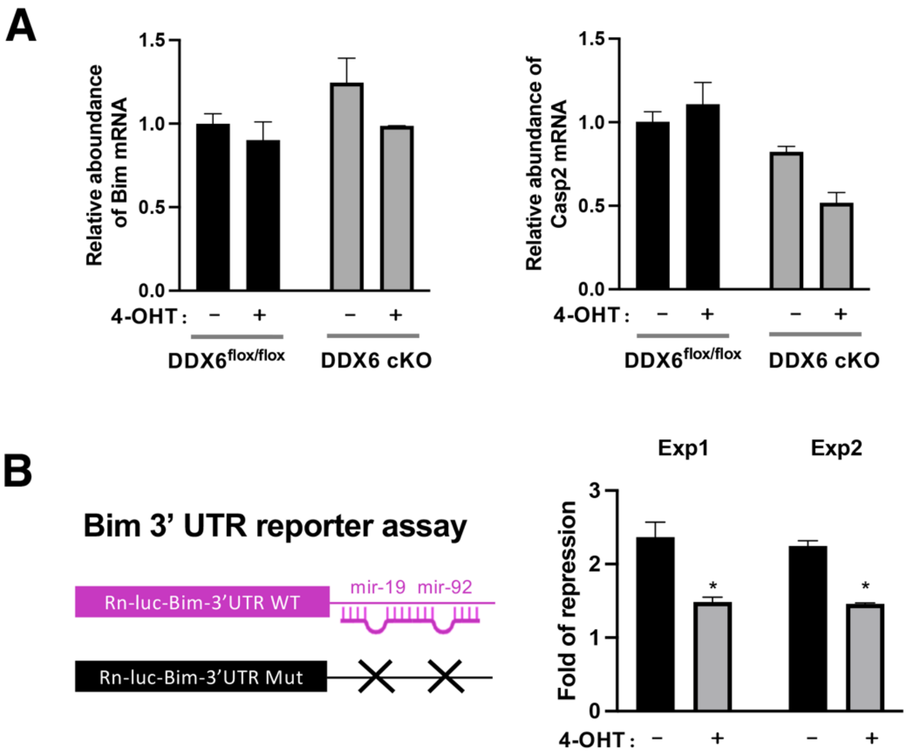

Figure S2. Additional evidence for miRNA silencing defects in DDX6 cKO mESCs. A. RT-qPCR analysis showed that no increase in Bim and Casp2 mRNA abundance relative to GAPDH mRNA as a control after DDX6 depletion. **B**. In DDX6 cKO mESCs, a Renilla luciferase (Rr-luc) reporter containing a fragment of the *Bim* 3′UTR with functional binding sites for miR-19 and miR-92 was compared to a reporter with a mutated *Bim* 3′UTR, with or without 4-OHT treatment. Firefly luciferase (Ff-luc) was used as an internal control. Data represent two independent experiments, each with three transfections. * $p < 0.05$.

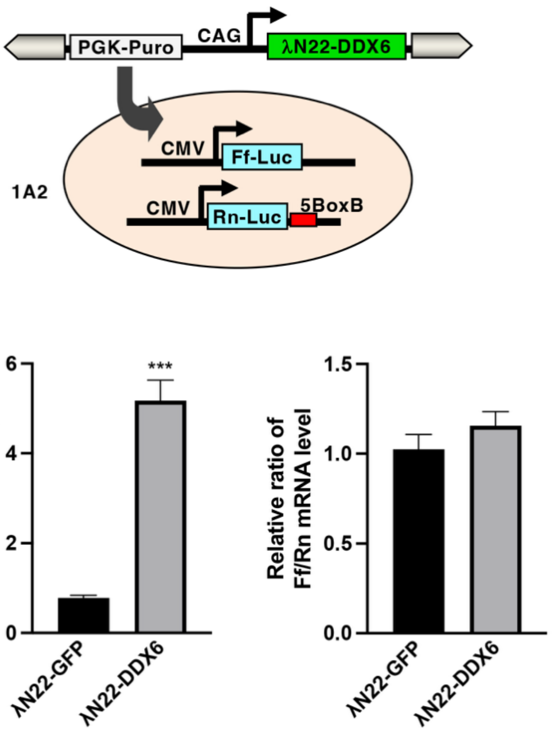

Figure S3. DDX6-mediated translational repression of a reporter gene. Tethering of λN22HA-DDX6 is sufficient to repress Rn-luc activity from a reporter containing five BoxB elements in the 3′UTR. Ff-luc was used as an internal control in 1A2 reporter cells. Rn-luc activity was suppressed by λN22HA-DDX6 without a corresponding decrease in relative mRNA abundance. mRNA levels of Rn-luc and Ff-luc were measured by RT-qPCR.

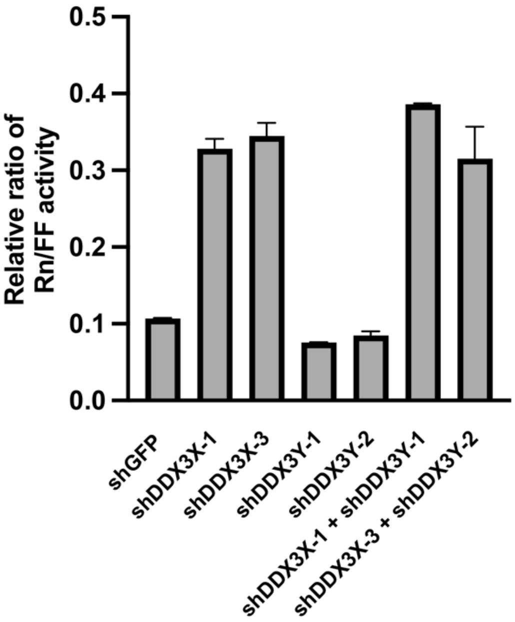

Figure S4. Contribution of DDX3Y to translational repression in mESCs. In 1D1 cell line carrying the λN22HA-DDX6 tethering reporter, knockdown of DDX3Y—either alone or in combination with DDX3X—using two different shRNAs had minimal impact on reporter activity compared to GFP control or DDX3X knockdown alone. Shown are the relative Rn-luc/Ff-luc activity ratios from stable 1D1 cell lines expressing individual or combined shRNAs.

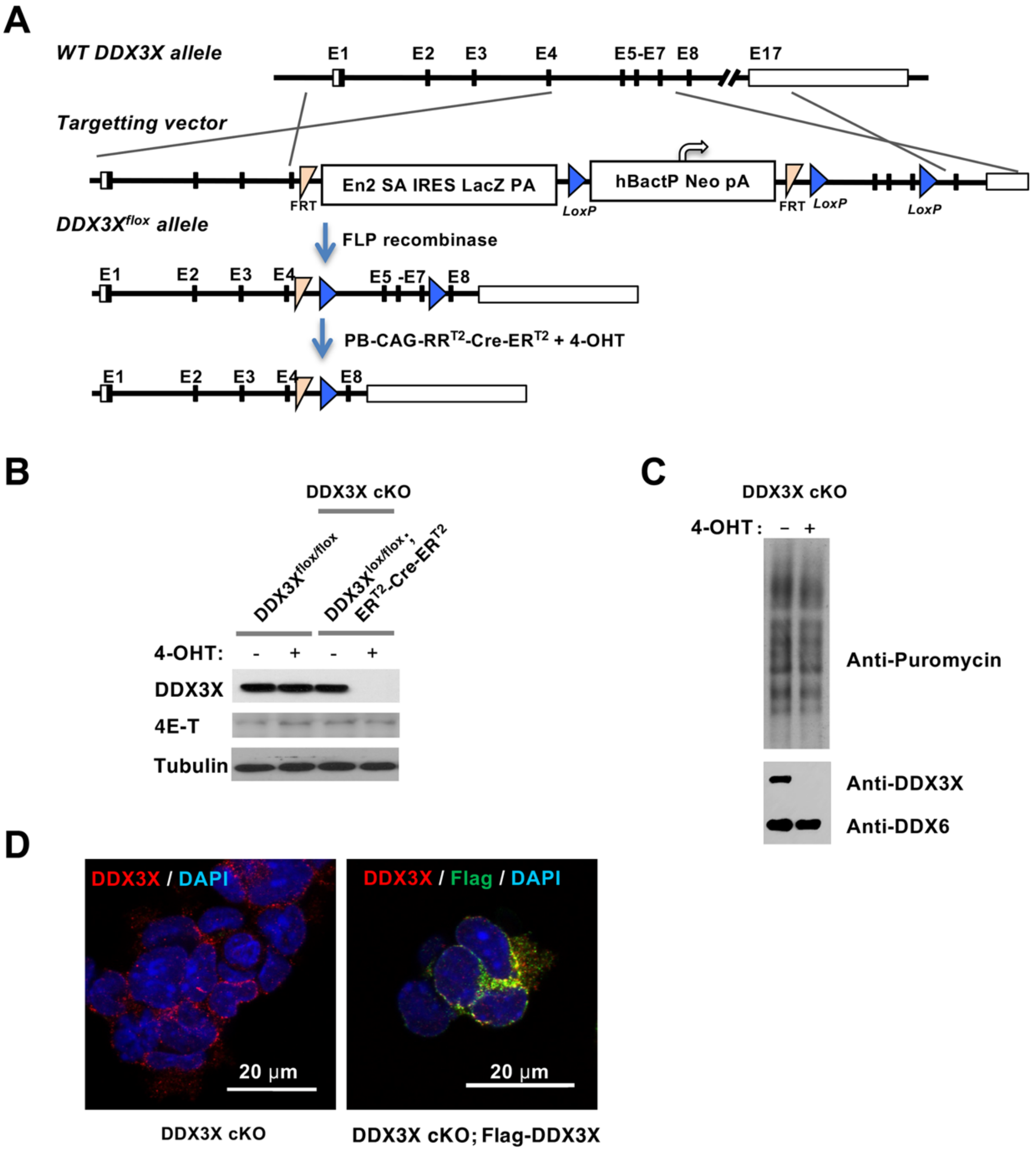

Figure S5. Generation of inducible DDX3X knockout mESCs. A. The wild-type locus and targeting vector for the mouse DDX3X gene are shown. Exons 5–7 are flanked by two loxP sites in the targeted DDX3X^{flox} allele. **B.** Western blot analysis confirmed efficient depletion of DDX3X two days after Cre activation. Protein levels of 4E-T and tubulin remained unchanged and served as loading controls. **C.** Pulse labeling of global protein synthesis with puromycin in DDX3X cKO cells. **D.** Subcellular localization of endogenous DDX3X and exogenous Flag-DDX3X proteins in mESCs. DDX3X proteins are broadly distributed throughout the cytoplasm.

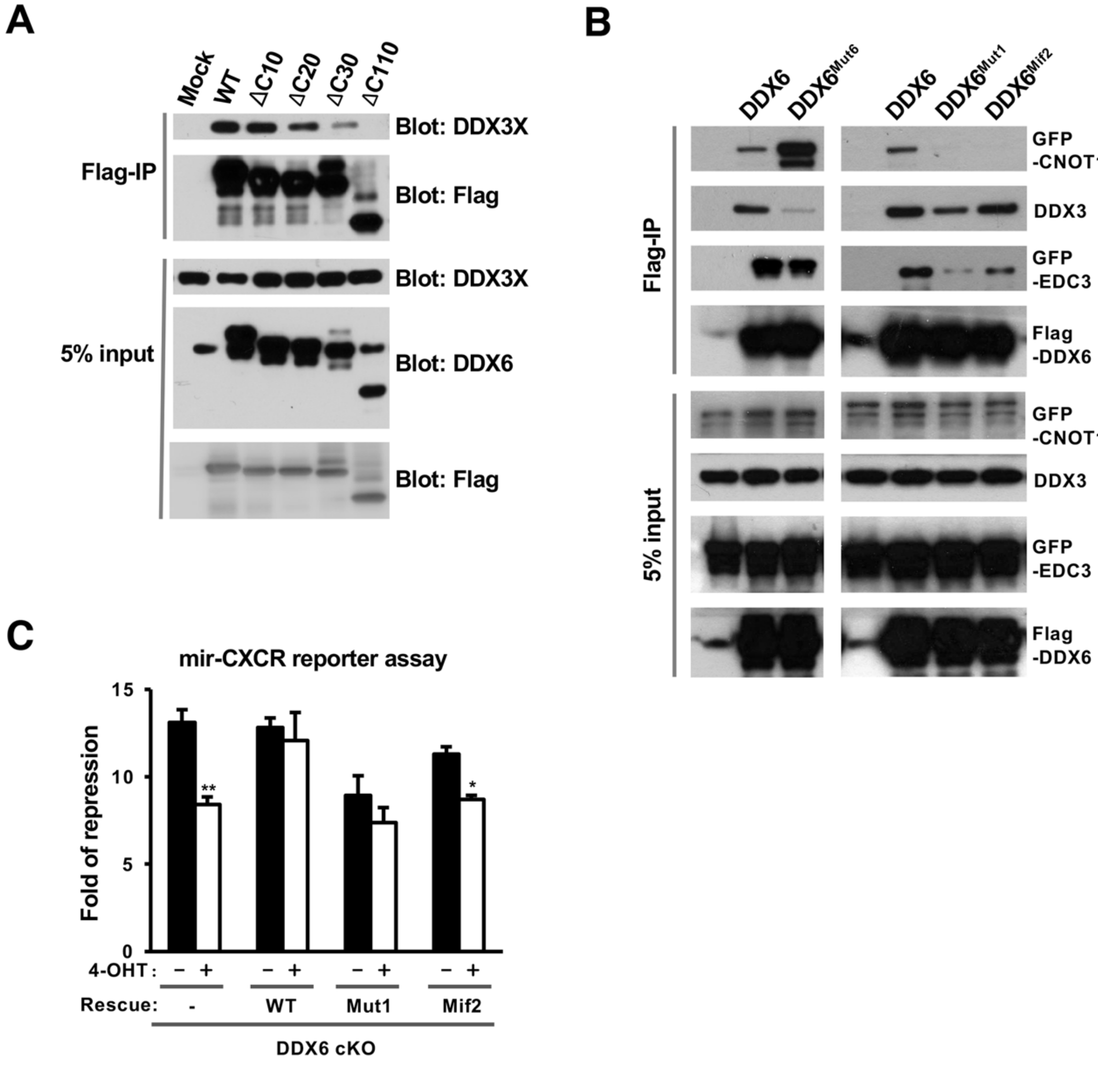

Figure S6. Characterization of a mutant DDX6 defective in DDX3X binding. A. The co-IP experiment mapped the C-terminal sequences of DDX6 required for DDX3X interaction in 293T cells. **B.** Comparison of the complex compositions between wild-type and mutant DDX6 proteins, showing that $DDX6^{Mut6}$ maintains interactions with other components in miRISCs, whereas $DDX6^{Mut1}$ and $DDX6^{Mif2}$ bind to DDX3X. **C**. mirCXCR reporter assays in DDX6 cKO mESCs genetically rescued by $DDX6^{Mut1}$ or $DDX6^{Mif2}$. All results are shown as means ± SEM. *$p < 0.05$, **$p < 0.01$, ***$p < 0.001$.

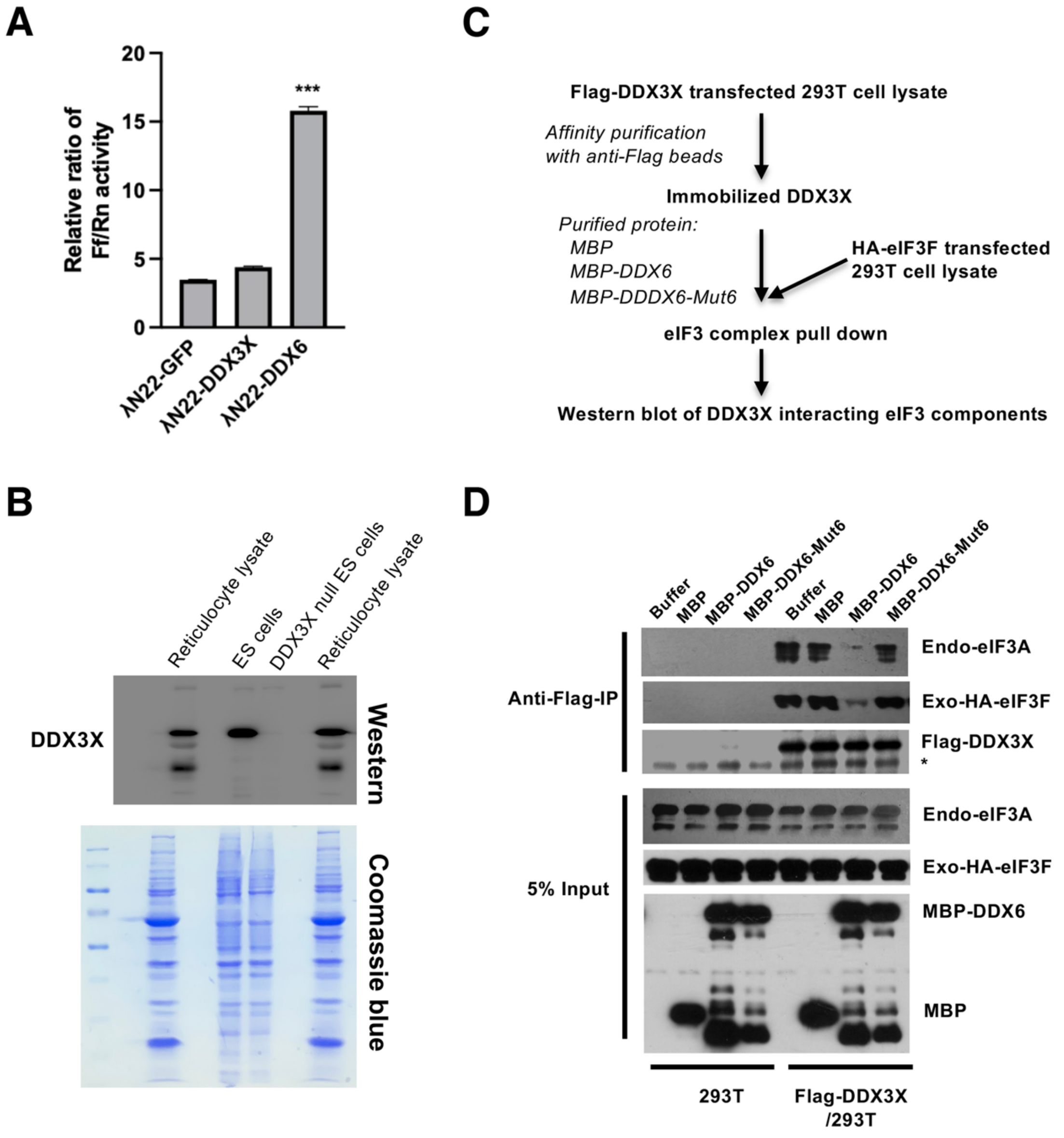

Figure S7. DDX3X is associated with translation machinery. **A.** Tethering of λN22-DDX3X was not sufficient to repress Rn-luc activity from a BoxB-containing reporter in the 1A2 mESC line. λN22-DDX6 served as a positive control. **B.** Western blot analysis confirmed the presence of DDX3X in commercially available reticulocyte lysate, with levels comparable to those in mESC lysate. Coomassie blue staining verified equal protein loading. **C**. Schematic of the experimental strategy to test whether DDX6 interferes with the interaction between DDX3X and the eIF3 complex. **D**. Endogenous eIF3A and exogenous HA-tagged eIF3F subunits were efficiently pulled down by Flag-DDX3X. As illustrated in **C**, recombinant wild-type MBP-DDX6 competitively inhibited DDX3X–eIF3 interaction, whereas the interaction-deficient mutant MBP-DDX6^{Mut6} failed to do so.
